# Enhancing the Quality of Service for Real Time Traffic over Optical Burst Switching (OBS) Networks with Ensuring the Fairness for Other Traffics

**DOI:** 10.1371/journal.pone.0161873

**Published:** 2016-09-01

**Authors:** Mohammed A. Al-Shargabi, Asadullah Shaikh, Abdulsamad S. Ismail

**Affiliations:** 1 College of Computer Science and Information Systems, Najran University, Najran, Saudi Arabia; 2 Department of Computer Science, Faculty of Computing, Universiti Teknologi Malaysia, Johar, Malaysia; West Virginia University, UNITED STATES

## Abstract

Optical burst switching (OBS) networks have been attracting much consideration as a promising approach to build the next generation optical Internet. A solution for enhancing the Quality of Service (QoS) for high priority real time traffic over OBS with the fairness among the traffic types is absent in current OBS’ QoS schemes. In this paper we present a novel Real Time Quality of Service with Fairness Ratio (RT-QoSFR) scheme that can adapt the burst assembly parameters according to the traffic QoS needs in order to enhance the real time traffic QoS requirements and to ensure the fairness for other traffic. The results show that RT-QoSFR scheme is able to fulfill the real time traffic requirements (end to end delay, and loss rate) ensuring the fairness for other traffics under various conditions such as the type of real time traffic and traffic load. RT-QoSFR can guarantee that the delay of the real time traffic packets does not exceed the maximum packets transfer delay value. Furthermore, it can reduce the real time traffic packets loss, at the same time guarantee the fairness for non real time traffic packets by determining the ratio of real time traffic inside the burst to be 50–60%, 30–40%, and 10–20% for high, normal, and low traffic loads respectively.

## Introduction

Optical Burst Switching (OBS) [1] network is the next generation of the optical Internet backbone infrastructure due to it’s attractive characteristics. OBS network is designed for better utility of wavelengths, to minimize the latency (setup delay), and avoid the use of optical buffers. In addition, the existing limitations of all optical networks such as the need of optical buffers are taken into consideration in the OBS network design, besides it supports the bursty traffic that could be generated from the upper level protocols or high level applications. Moreover, OBS network uses one-way reservation scheme that supports sending high data rate and low latency traffics.

Accordingly, all these features indicate that the OBS network can be fundamental infrastructure of next generation optical Internet. Stability in the Internet network performance is a significant issue. It depends on the Quality of Service (QoS) that should be guaranteed to support thigh priority traffic categories, such as Constant Bit Rate (CBR), Variable Bit Rate (VBR), and depends on fairness among other traffic types that should be also ensured. In this paper we propose a novel Real Time Quality of Service with Fairness Ratio (RT-QoSFR) scheme to adapt the burst assembly parameters according to the real time traffic QoS needs, and simultaneously to ensure the fairness among other traffic types for enhancing the real time traffic QoS requirements over OBS network.

RT-QoSFR can guarantee the entire delay of OBS network such that it does not exceed the MaxCTD parameter value in the real time traffic. Furthermore, it can reduce the real time traffic packets loss and guarantee the fairness for non real time traffic packets. Moreover, RT-QoSFR guarantees the stability in the performance of the network, the delay requirements, and ensures the fairness between real time traffic and non real time traffic, which provides a better QoS.

The proposed schemes have been studied using the simulation model with two types of traffic (CBR, VBR), four values of MaxCTD, two value of burst size, and two different topologies (the simple OBS topology, and the NSFNET topology). The objective of these scenarios is to demonstrate the possibility of the proposed scheme to work under various conditions.

The remainder of the paper is structured as follows. Section presents related work. Section proposed RT-QoSFR scheme is introduced while Section explores simulation model, results, and discussions. Finally, Section provides the conclusions and identifies directions of future work.

## Related Work

In OBS network, the exchange of data between the source and destination transfers through several sub-processes, which will either add some extra delay time or cause some data loss. These sub-processes can affect the QoS requirements and traffic contract in real time compressed traffic.

Previously, several schemes in several OBS sub-process have been proposed to guarantee the QoS for the high priority traffic. Each scheme tried to achieve guarantee of QoS from different aspects. However, guaranteeing the QoS for real time compressed traffic over OBS has not been fully achieved.

In the burst assembly sub-process, the hybrid time-and-threshold-based scheme [2] [3] has been proposed as a scheme to balance the time and size of the data burst to provide better QoS. In this scheme, the burst is created either when the timer reaches to the maximum value of Time out (*T*_*out*_) or when the number of bytes reaches the maximum value of Burst minimum (*B*_*min*_). Thus, this scheme is currently assumed to be the default burst assembly scheme. It combines the benefits of both the time-based burst assembly and the threshold-based schemes. However, the hybrid scheme is not considering the real time traffic delay requirements in the case of low network traffic load. Where, the real time traffic will be forced to wait until the timer reaches to its maximum value, then it is assembled and sent to the destination. This delay affects the real time traffic delay requirements.

On the other hand, Learning-based Burst Assembly (LBA) [4] proposed an algorithm for adapting the burst assembly time based on the observed loss pattern in the network. It employs an algorithm model that uses learning automata, which probes the loss in the network periodically and changes the assembly time at the ingress node to a favourable one. The selecting of an assembly time parameter value is depending on the loss measured over the path using the linear reward-penalty approach. The advantage of LBA scheme is that it can reduce the burst loss probability as compared to the other adaptive assembly mechanisms. On the other hand, this scheme does not consider the traffic QoS delay requirements or needs. Thus, LBA cannot be used for real time traffic.

In the contention resolution sub-process, burst segmentation [5] is a contention resolution that prefers to lose a few packets from the contending burst instead of losing the whole burst. When a contention occurs between two bursts, the overlap between bursts will be dropped. The main advantage of the tail dropping is the in-sequence delivery of the packets at the destination.

However, aggregating the burst from the high priority traffic only will increase the average of the loss of its packets. Therefore, a combination of several traffic types can be achieved in one burst. Researchers in [5] [6] [7] proposed a prioritized contention resolution method, in which the edge node combines packets of different traffic priority into the same burst, where the lower priority traffic packets are aggregated at the tail of the burst, head of the burst, or middle of the burst. Accordingly, a complete isolation of the highest priority traffic (e.g., real time traffic) can be achieved, which will provide much better QoS for the highest priority traffic. However, the ratio of the highest priority traffic in the burst is very significant for providing QoS for this type of traffics. Moreover, this scheme does not guarantee the QoS delay requirements because of the assembly process does not consider the delay requirements while aggregating the bursts.

In the signalling sub-process, OBS network uses a one-way reservation mechanism to allocate the resources where the control packet proceeds the data burst in an amount of time called the ‘offset time’. The offset time is the amount of time required by the control packet to successfully allocate the resources. In case the offset time is not enough to allocate all the resources in the destination path, the data burst is dropped. The researcher in [8] proposed the offset-based OBS QoS mechanism to ensure that higher priority classes have a greater chance to allocate the resources than the lower priority classes. The offset-based OBS QoS mechanism adds an additional offset time between the control packet, and data burst based on the priority of service class. Thus, higher priority bursts gain an additional time to increase the reservation possibility. As a result, the higher priority bursts segment the data wavelengths with leaving gaps between the segments. Therefore, lower priority bursts tend to reserve only the gaps left by the higher priority bursts. Even though the offset-based OBS QoS mechanism provides a higher reservation probability to the higher priority bursts, it also causes the higher priority bursts to wait for a long time prior to being served. On the other hand, the short low priority bursts have a lower burst loss probability than the longer low priority bursts, as they have a higher probability to fit into the gaps. This is contradictory to the control overhead which is low when the low priority bursts are high. Furthermore, the starvation of the low priority classes is still possible if the offered traffic load of the high priority bursts is not controlled.

Song H, Brandt-Pearce M, Xie T, Wilson SG developed an innovative concatenation scheme that works in two TIER; (1) inner code, and (2) outer code. Inner code is being constrained code based on Total Impairment Extent Rank (TIER) while outer code being a low-density parity-check (LDPC) code. In order to avoid the affect of Amplified Spontaneous Emission (ASE) noise on system performance for long-haul fiber-optic communication systems, a novel TIER is developed to prevent deterministic physical impairments and the ASE noise. This TIER constrain code from coding scheme and restrain physical impairments, including linear effects and nonlinear effects [9].

While, Song and Pearce [10] introduced model that works on several channel effects, fiber loss, and frequency chirp, which are omitted in the literature. Furthermore, this model also works on coefficients that capture Inter Symbol Interference (ISI) and several other characteristics.

Same authors also worked on another model that works on several channel effects, fiber loss, frequency chirp, optical filtering, and photo detection which are omitted in the literature. The model offers an agreement with obtained results by split-step fourier simulation. Moreover, this model covers several characteristics such as ISI, inter channel interference, self-phase modulation, intra channel cross-phase modulation (XPM), intra channel four-wave mixing (FWM), XPM, and FWM to improve the system performance [11].

The authors in [12] presented the case study regarding big data stream mobile computing. It is detail study of traffic offloading, reconfiguration of network data, and big data stream mobile computing. The source discusses the case study on StreamCloud.

OBS is considered as an optical network technique that allows wavelength-division multiplexing (DWDM) and in this regard, the method of Volterra series transfer function (VSTF) is presented that state characteristic coefficients to record intersymbol interference (ISI), self phase modulation (SPM), intrachannel cross phase modulation (IXPM), intrachannel four wave mixing (IFWM), cross phase modulation (XPM) and four wave mixing (FWM), to classify the influence of these components on the system output [13]. Furthermore, a discrete-time input-output model is introduced for single channel multipulse multispan fiber-optic communications systems based on the VSTF method. This model created an agrement with SSF method and its use has been shown by new coding scheme to prevent the development of intrachannel interferences [14].

There is huge delay issues with real-time cloud services and in order to address these issues, Shojafar M, Cordeschi N, Baccarelli E [15] proposed an energy-efficient adaptive resource scheduler for Networked Fog Centers (NetFCs). The purpose of scheduler is to make full use of states of the TCP/IP connections, to make great as possible the overall communication while meeting the QoS requirements.

Alternatively, an adaptive offset time scheme in OBS network [16] proposed an extra offset time which is assigned to the bigger burst size to achieve isolation in the burst. However, the extra delay that will be added to the packets’ delay causes effect on the QoS delay for those packets.

It is clear from the open literature that the preceding mechanisms are able only to guarantee either the delay requirements or data loss requirements. Furthermore, the fairness among the traffic type is absent in these schemes. Thus, a new scheme that guarantees both requirements with ensuring the fairness among the traffic types is required. This paper proposes a new scheme called Real Time Quality of Service with Fairness Ratio (RT-QoSFR).

## Real Time Quality of Service with Fairness Ratio (RT-QoSFR) Scheme

In this section, a novel Real Time traffic—QoS with Fairness Ratio (RT-QoSFR) scheme based on a new burst assembly algorithm is introduced. RT-QoSFR adapts the burst assembly parameters (the traffic ratio inside the burst, (*T*_*out*_) according to the traffic requirements and load so as to reduce the real time traffic packets loss, and at the same time, it guarantee the fairness for non real time traffic packets. The traffic ratio inside the burst is adapted based on a statistical study that has been carried out to find the best ratio (fairness ratio) for the real time traffic packets inside the data burst in various network traffic loads. Furthermore, *T*_*out*_ is adapted based on the most appropriate value to meet the end to end delay requirements of real time traffic. As a result, RT-QoSFR promises the required QoS that creates stability in the performance of the network, ensures the fairness between real time traffic, and non real time traffic, which all collaborate to provide a better QoS.

The ratio of the high priority traffic in the burst is a very important issue for providing the QoS for this traffic. For example, if the edge node aggregates 10% of the burst as a high priority traffic and 90% as a low priority traffic, this ratio could reduce the loss of the high priority traffic. Conversely, it will increase the overall loss in the core node and then affect the performance of the network. This loss is due to the large number of burst that will be aggregated in the edge node which will increase the overall loss at the core node. Additionally, if the edge node aggregates 90% of the burst as a high priority traffic and 10% as a low priority traffic, this ratio could reduce the loss in the core node due to the small number of burst that will be aggregated but will also increase the loss of the high priority traffic. Thus, the ratio of the real time traffic inside the burst is essential to reduce the real time traffic packets loss rate. Consequently, this section is divided into two parts that are the statistical study to find the fairness ratio for the real time traffic packets inside the data burst and the design RT-QoSFR scheme.

### Statistical Study to Find the Fairness Ratio for the Real Time Traffic in the Data Burst over OBS Network

This statistical study has been carried out to find the best ratio (fairness ratio) for the real time traffic packets against the non real time traffic packets in various network traffic loads. The statistical study is based on the Significant difference (Sd) factors between the network’s Over all loss (*O*_*l*_*oss*) and the Real time traffic loss (*R*_*l*_*oss*).

The significant difference factors have been found using a simulation model which deals with two types of real time traffic (CBR and VBR), two values of burst size, two different topologies (four Nodes OBS topology, National Science Foundation Network NSFNET topology), incremental load traffic rate, and ten ratio values for real time traffic (10-100%). As a result, ten Sd values have been produced for each case in the study as follows:

$$\begin{array}{ccc} for\;x=10,20,30,..,100 & & \\ sd\left(x\right)=∥O{\left(x\right)}_{drop}-R{\left(x\right)}_{drop}∥ & \end{array}$$ (1)

Where *x* determines the real time traffic ratio in the data burst. In this equation, the value of *x* will be replaced, ten times, with the real time ratios in the data burst and stored in the Sd group. Consequently, based on these results, mathematical equations to find the range of the best ratio for real time traffic in the burst that can guarantee the fairness between real time traffic and non real time traffic have been found. The mathematical equations aim to find the lowest two minimum ratio in Sd; the Lowest ratio (*Low*_*ratio*_), and Second Lowest ratio (*SLow*_*ratio*_). Firstly, the equation that aims to find the minimum Sd value (*Low*_*value*_) is as follows:

$$\begin{array}{c} Low_{value}=min\left\{sd\right\} \end{array}$$ (2)

Subsequently, it aims to find out the ratio of this value *Low*_*ratio*_ by searching in all the Sd group ratio and assign it to *Low*_*ratio*_ as follows:

$$\begin{array}{ccc} for\;i=10,20,30,..,100 & & \\ Low_{ratio}=\;i,\;if\;Low_{value}=Sd\left(i\right) & \end{array}$$ (3)

The second minimum Sd (*SLow*_*ratio*_) can be found by using a temporary group of elements *Sd*_*temp*_ that contains all Sd except *Low*_*ratio*_:

$$\begin{array}{c} \left\{Sd_{temp}\right\}=\left\{Sd\right\}/Low_{ratio} \end{array}$$ (4)

Therefore, *SLow*_*value*_ will be the minimum value of the new temporary group *Sd*_*temp*_ as follows:

$$\begin{array}{c} SLow_{value}=min\left\{Sd_{temp}\right\} \end{array}$$ (5)

Subsequently, this value can be used to find *SLow*_*ratio*_ by searching in all the *Sd*_*temp*_ group ratio and assigning it to *SLow*_*ratio*_:

$$\begin{array}{ccc} for\;i=10,20,30,..,100 & & \\ SLow_{ratio}=\;\left\{i,\;if\;SLow_{value}=Sd_{temp}\left(i\right)\right\} & \end{array}$$ (6)

Thus, after getting the values of *Low*_*ratio*_ and *SLow*_*ratio*_, the *Avg*_*ratio*_ of the real time traffic in the burst will be in the range between *Low*_*ratio*_ and *SLow*_*ratio*_ based on the network traffic load according to:

$$\begin{array}{c} Avg_{ratio}≬Low_{ratio}\wedge SLow_{ratio} \end{array}$$ (7)

The objective of this study is to demonstrate the exact ratio that can ensure the real time traffic loss requirements and to the fairness for the non real time traffic loss requirements. Moreover, this study aims to make sure that the accuracy of this ratio is capable to work under various conditions such as different type of real time traffic, various values of burst size, or various design of the topology.

### The Statistical Study Simulation Results and Discussions

In this section, the equations in the previous section have been implemented in the simulation model that have been used to find the fairness ratio. The configuration of the simulation models is divided into two parts: (1) the OBS network configuration and (2) the real time traffic configuration. In the real time traffic configuration, CBR traffic and VBR traffic trace files have been created with incremental load. The results show that the aggregation process can be categorized based on the traffic load into three categories, which are the low, normal, and high loads. In the high load, the results show that the best ratio for real time traffic in the burst that can guarantee the fairness between real time traffic and non real time traffic is between 50% and 60%.

**Fig 1:**
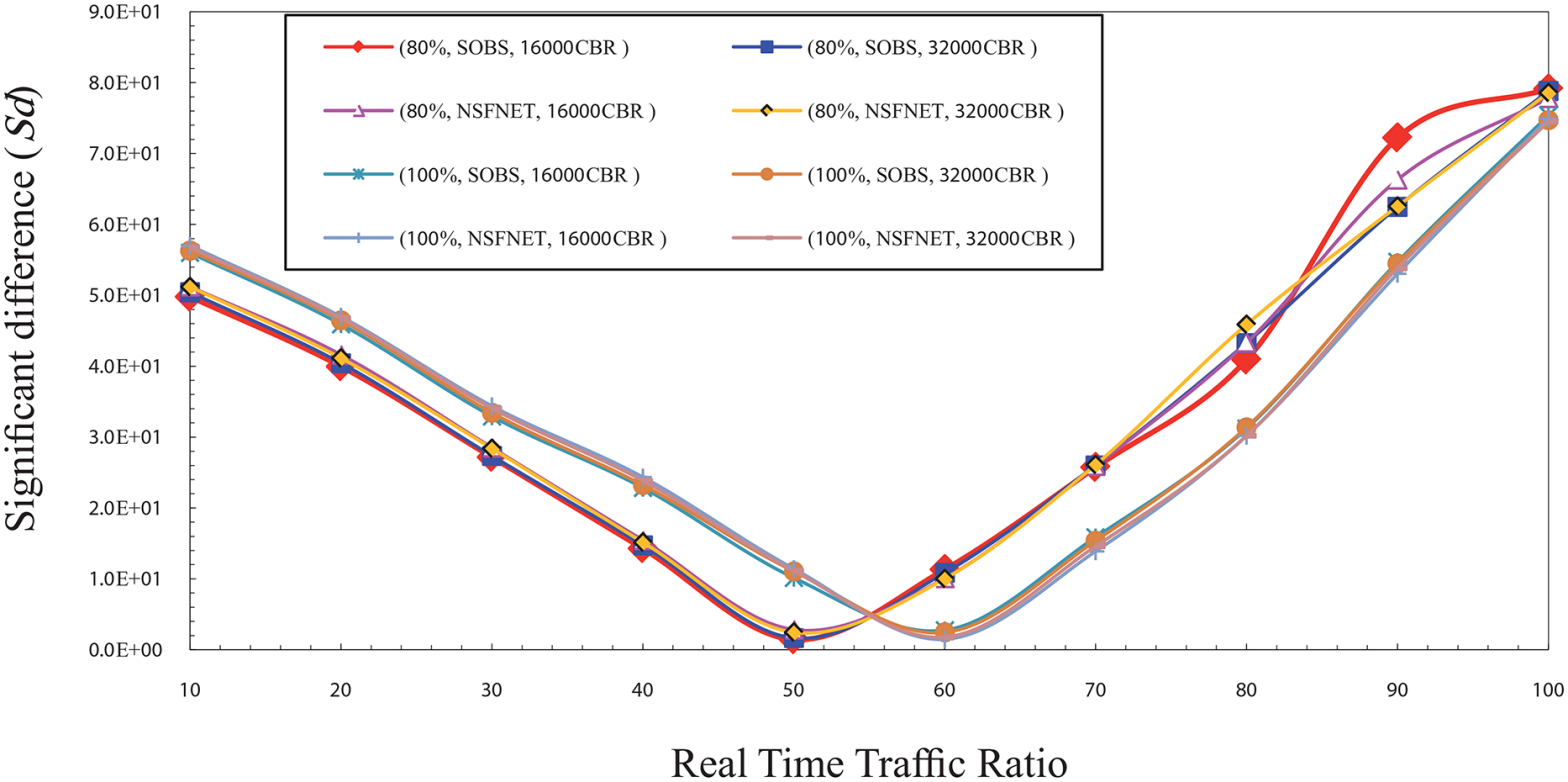
The significant difference (Sd) factor values in the high traffic load for CBR traffic.

**Fig 2:**
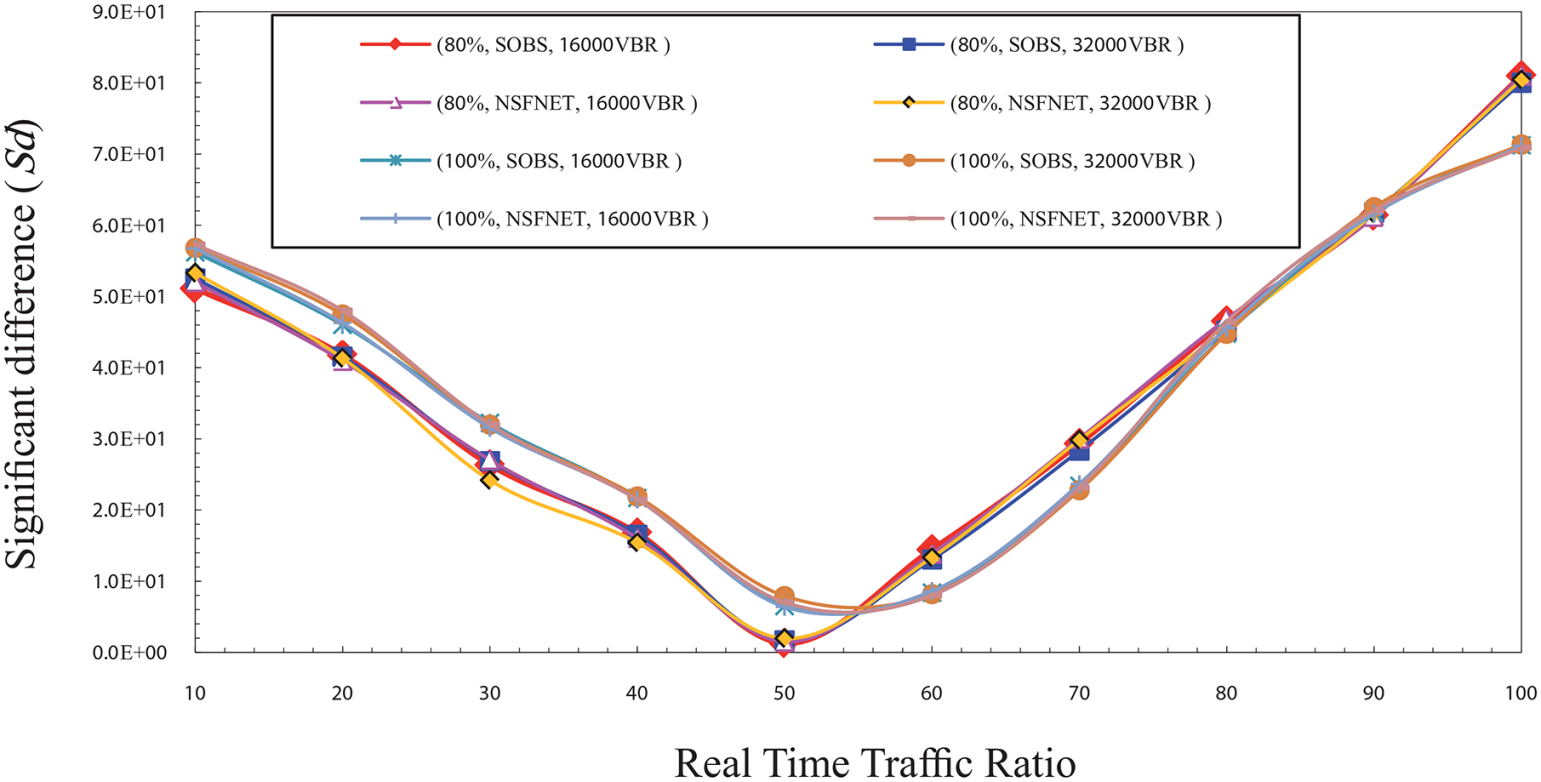
The significant difference (Sd) factor values in the high traffic load for VBR traffic.

Figs 1 and 2 show the significant difference (Sd) factor in the high traffic load for CBR and VBR traffic. Sd has been studied as shown in the figs with several factors that are the traffic type (CBR, and VBR), burst size (16000 Kbyte, 32000 Kbyte) [17], network topology layout (four nodes OBS, NSFNET), and traffic load (80%, 100%) of the total bandwidth which is 1 Gbps. The results show that the best ratio is ranging from 50% to 60% based on the network traffic load value.

It is noted that the value of Sd gradually decreases from the ratio of 10% to 50%. This is because of the numerous number of data burst that is created and sent to the core network due to the small ratio of real time traffic in each burst. It leads to an increase the number of data burst needed to send all the real time traffic available in a certain period. This large number of burst increases the rate of burst loss in the core node which creates a discrepancy between the value of the network overall loss and the real time traffic packets loss. Thus, it can be noted that the Sd value goes high in the case of ratio of 10% and reduces with the increase of the ratio of real time traffic in the burst which reduces the rate of burst loss until it reaches to the ratio 50%.

**Fig 3:**
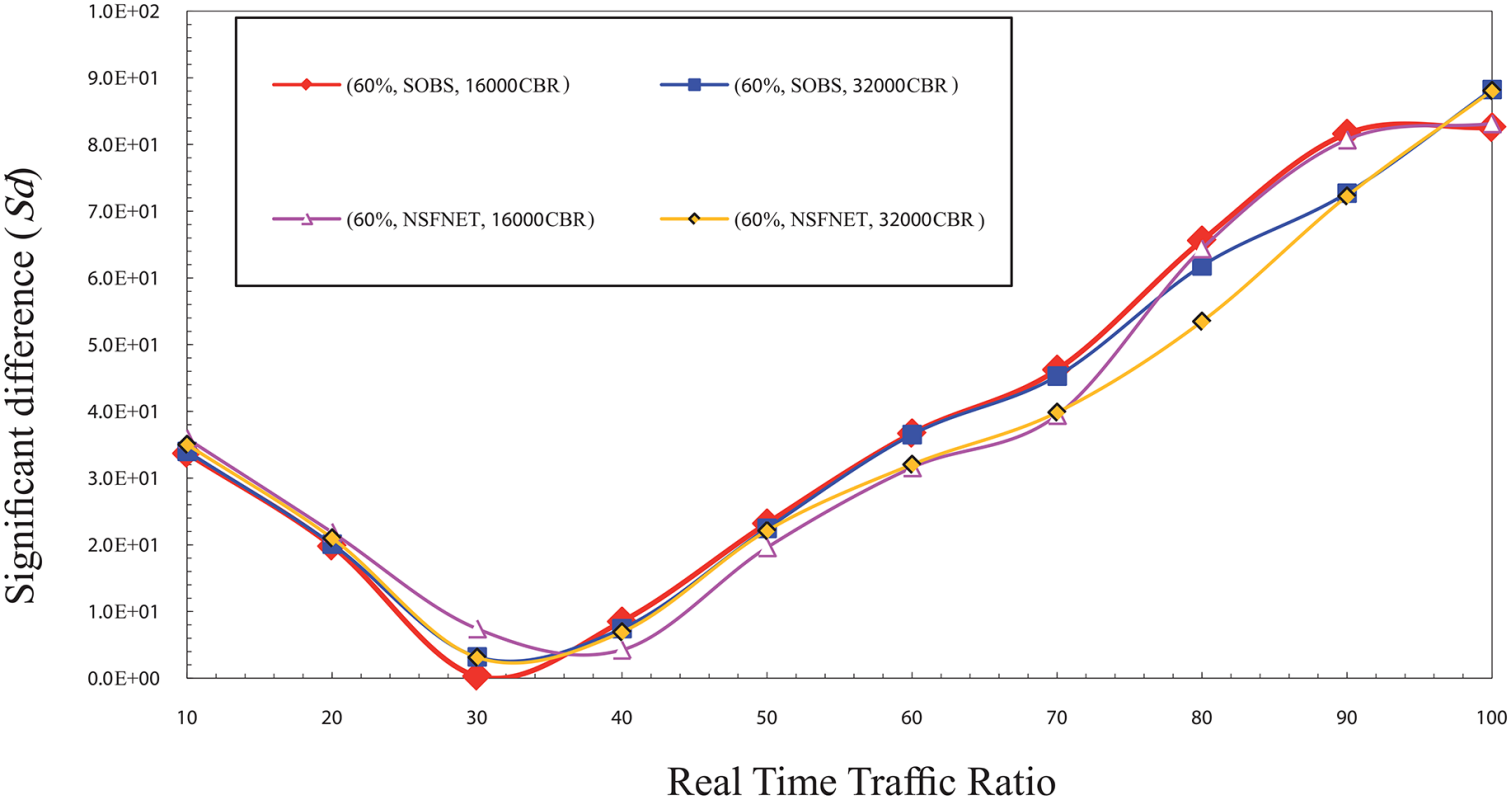
The significant difference (Sd) factor values in the normal traffic load for CBR traffic.

**Fig 4:**
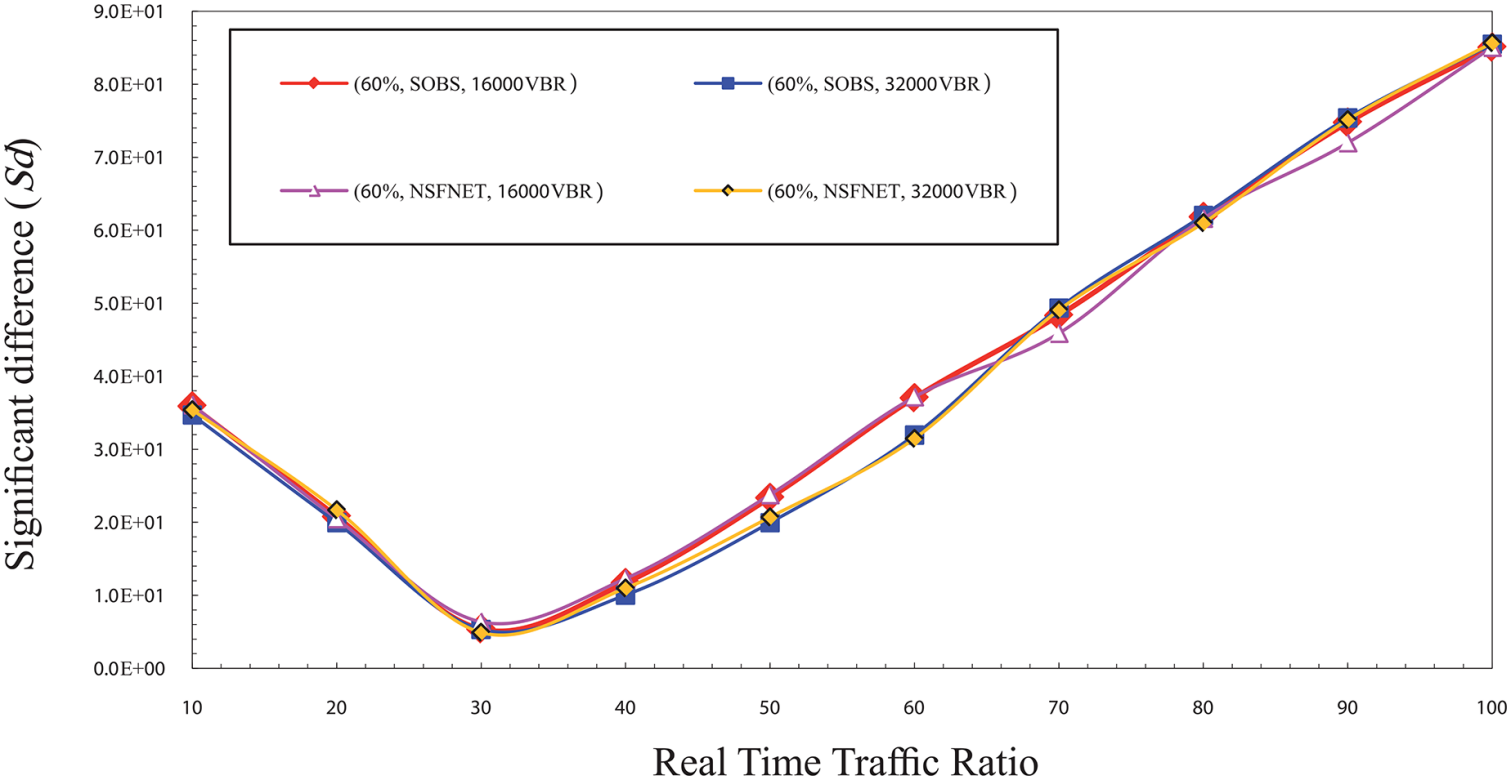
The significant difference (Sd) factor values in the normal traffic load for VBR traffic.

In contrast, it is noted that the value of Sd gradually increases from 60% up to 100%, due to the rise of the rate of real time traffic packets loss caused by its high ratio in the burst and the low rate of overall loss which is created by the low number of bursts. Therefore, the Sd value is high at these ratios and getting a raise with the increase of the ratio of real time traffic in the burst. It causes an increasing of the rate of real time traffic packets loss until it reaches to the ratio 100%. In the normal load, the results show that the best ratio for real time traffic in the burst that can guarantee the fairness between real time traffic and non real time traffic is between 30% and 40%. Figs 3 and 4 show the Sd factor in the normal traffic load for CBR and VBR traffic. The Sd has been studied using same factors that is used in the high traffic load as mentioned above.

In normal load case the value of Sd is low for the low ratios (10–50%) and high for the high ratios compared with the high load traffic case. This variation is due to the traffic load which increases the rate of traffic load in the high load case; while the rate of real time traffic packets loss is similar to both cases which make the Sd value goes high in the high traffic load case. In contrast, for the high ratios from 50% up to 100%, it is noted that the value of Sd is higher than the values of the low ratios due to the decrease of overall rate loss. In this case, the rate of real time traffic packets loss is similar to both normal and high traffic load case, which makes the Sd value higher in the normal traffic load case.

**Fig 5:**
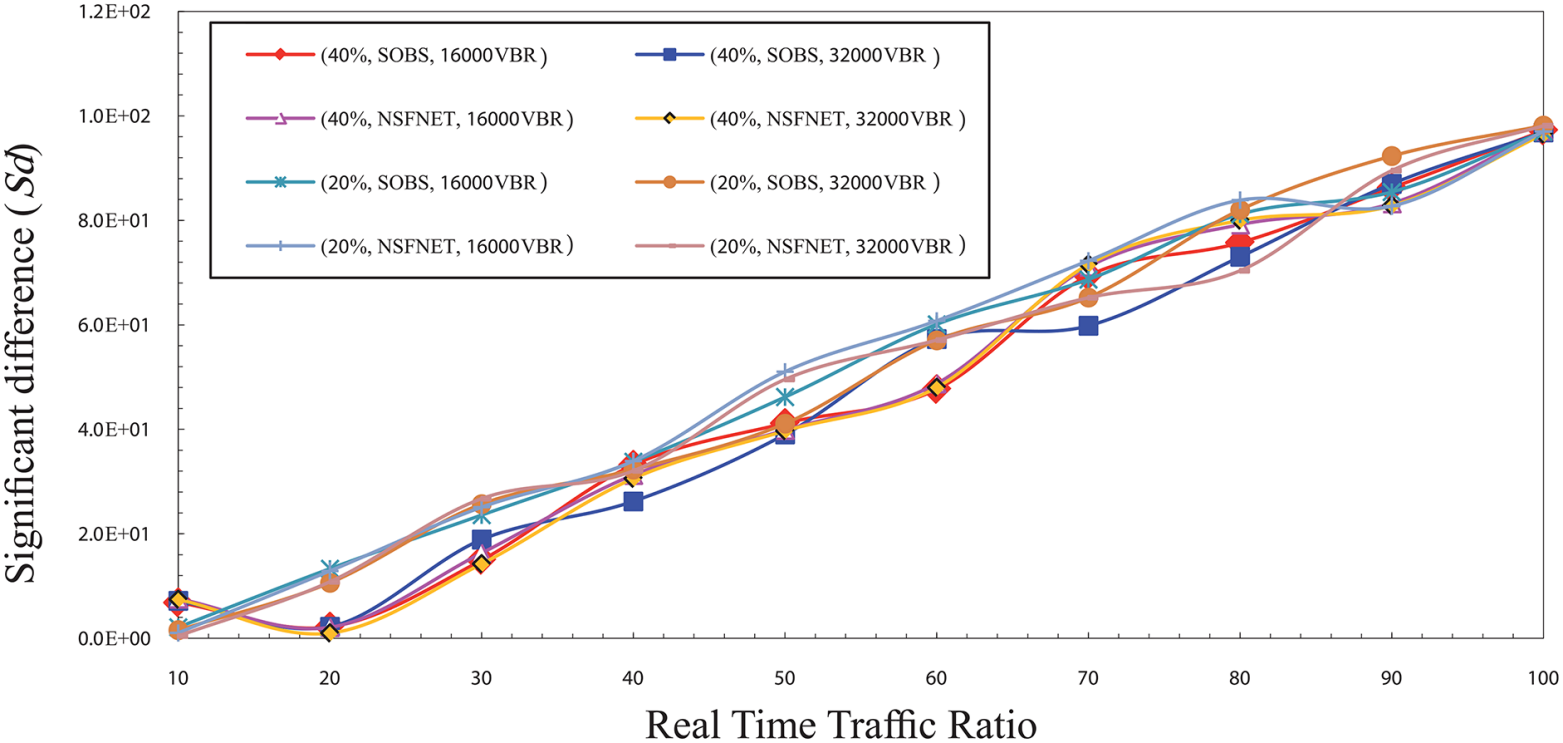
The significant difference (Sd) factor values in the low traffic load for CBR traffic.

**Fig 6:**
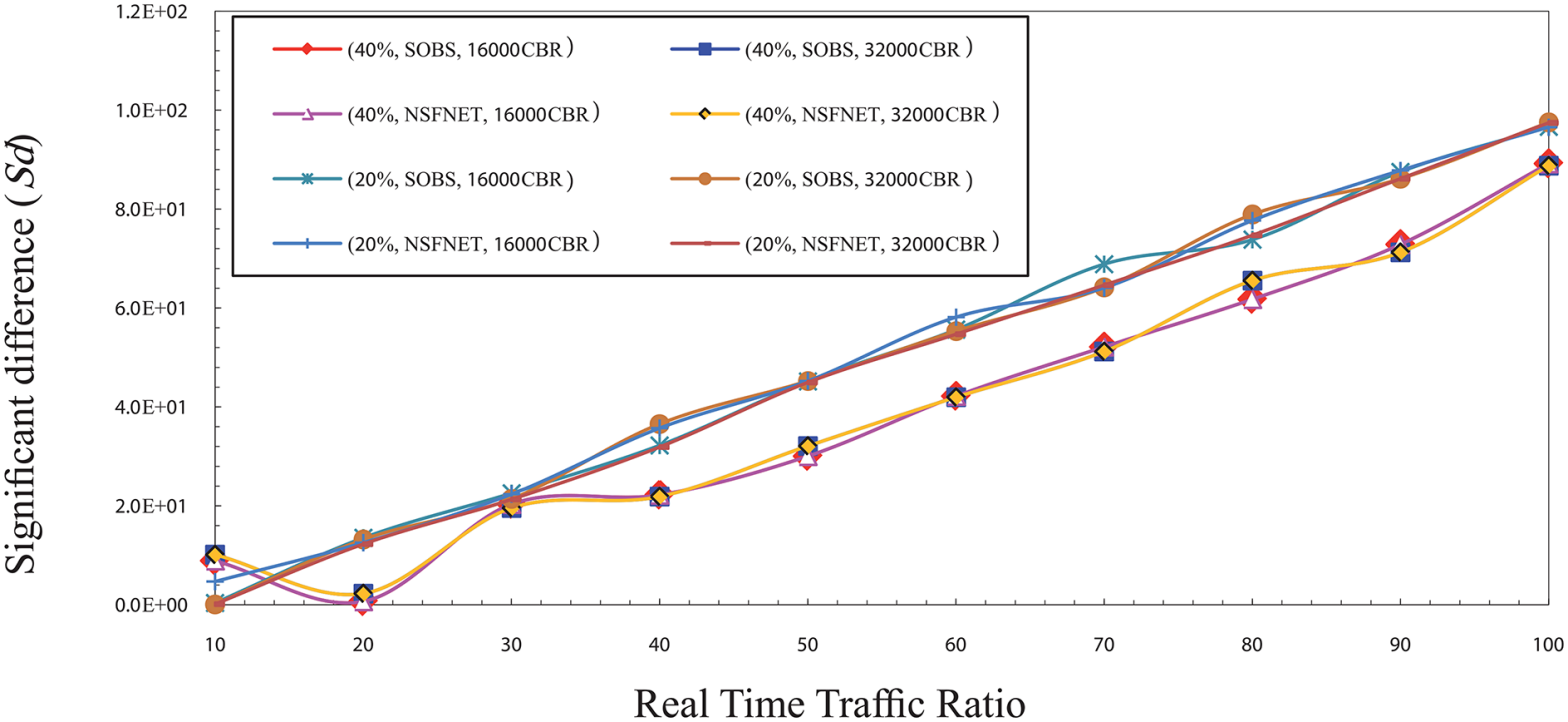
The significant difference (Sd) factor values in the low traffic load for VBR traffic.

In the low traffic load, the findings show that the best ratio for real time traffic in the burst that can guarantee the fairness between real time and non real time traffic ranges between 10% and 20%. Figs 5 and 6 show the significant difference (Sd) factor in the low traffic load for CBR and VBR traffic. Similarly, same factors are used for the cases mentioned above. It can be observed that the lowest Sd values are within the ratios 10% and 20%, which make them the best ratios for the real time traffic in the burst to guarantee the fairness concept. This finding is a result of the low traffic load which decreases the loss rate of overall traffic loads. In this case, the rate of real time traffic packets loss is similar in all cases which make the Sd value low in the low traffic load case.

On the contrary, it is noted from Fig 5 that the value of Sd is higher than the values in the two previous cases due to decrease of overall rate of loss. While the rate of real time traffic packets loss is similar in both cases which leads to a high Sd value in the low traffic load case.

In the following section, the design of RT-QoSFR scheme is discussed, therefore, Table 1 summarises all notations that are used in equations.

**Table 1:** Examples of local and global invariants.

Symbol	Full Form
*A*_*time*_	Aggregation Time
*BA*_*time*_	Burst Assembly time,
*B*_*max*_	Maximum Burst Length
*B*_*size*_	Burst Size
CBR	Constant Bit Rate
LBA	Learning-Based burst Assembly
MaxCTD	Maximum Cell Transfer Delay
*Max*_*delay*_	Maximum Delay
MBS	Maximum Burst Size
*M*_*Value*_	Membership Value
NCBS	Non-Composite Burst Segmentation
*NRTT*_*Bytes*_	Non Real Time Traffic Average
NRT-VBR	Non Real Time-Variable Bit Rate
NSFNET	National Science Foundation Network
OBS	Optical Burst Switching
*O*_*loss*_	Over all loss
OT	Offset Time
*P*_*delay*_	Propagation Delay
PT	Processing Time
QoS	Quality of Service
*R*_*loss*_	Real time traffic loss
RT-ABA	Real Time traffic Adaptive BurstAssembly
*RTT*_*avg*_	Real Time Traffic Average
RT-VBR	Real Time- Variable Bit Rate
Sd	Significant Difference
ST	Switching Time
*T*_*out*_	Time Out
*T*_*time*_	Total Time
VBR	Variable Bit Rate
*H*_*avg*_	Ratios of real time traffic inside the burst for high traffic load parameters
*Low*_*avg*_	Ratios of real time traffic inside the burst for low traffic load parameters
*N*_*avg*_	Ratios of real time traffic inside the burst for normal traffic load parameters
*A*_*range*_	The maximum value of the membership value

### The Design of RT-QoSFR scheme

RT-QoSFR scheme works inside the optical network edge node to classify both traffic load and traffic type. RT-QoSFR scheme differentiates the data traffic based on the destination into different queues. In each destination queue, RT-QoSFR also differentiates the data traffic based on the traffic type into real time traffic or non-real time traffic queue. RT-QoS scheme employs two timers to aggregate the data; a default internal timer, and a separate timer on the real time traffic queue. The real time traffic timer is used to calculate the appropriate burst assembly time values *T*_*out*_ based on the maximum packet transfer delay value. The timers are separated due to the needs for a differentiated service, as well as to make the default internal timer works for all traffic types in case of no real time traffic. RT-QoS scheme starts with the first phase to identify the network traffic type and traffic load. RT-QoS scheme studies and analyzes the network load in each second through the following steps: it gets the summation of the Aggregation time for each burst (*A*_*time*_) every one second and assigns it as a Total time value (*T*_*time*_) according to Eq (8):

$$\begin{array}{c} T_{time}=\sum_{time=0}^{1\;sec}A_{time} \end{array}$$ (8)

Then, the summation of the Burst size (*B*_*size*_) is converted into bits and assigned to the Temporary size *Temp*_*size*_ variable. Next, RT-QoS checks the *T*_*time*_ value, if it becomes 1 second, it will assign the *Temp*_*size*_ to the total burst size *T*_*s*_*ize*, otherwise, it continues the counting. Eq (9) aims to find the total number of bits that have been sent in one second as follows:

$$\begin{array}{c} \left\{ \begin{array}{cc} T_{size}=Temp_{size}, & if\;T_{time}=>1sec\\ Temp_{size}=\sum B_{size}\times 8, & if\;T_{time}=>1sec \end{array}\right. \end{array}$$ (9)

After getting the number of bits in one second by using Eq (2), it will find the network traffic load average, *L*_*avg*_:

$$\begin{array}{c} L_{avg}=\{\begin{array}{cc} \left(\frac{T_{size}}{Bw}\right)\times 100_{,} & if\;T_{time}\geq 1sec\\ L_{avg}, & if\;T_{time}<1sec \end{array} \end{array}$$ (10)

where, Bw is the network bandwidth, and Lavg determines the network traffic load rate per 1 second. This equation will find the current network traffic load rate per 1 second compared with the bandwidth. Then, based on the traffic type, the phase two will start working; where one of the timers will be activated as in Eq (4).

$$\begin{array}{c} \left\{ \begin{array}{cc} Use\;\;RTT_{timer} & if\;Traffic\;\;type=RTT\\ Use\;\;InternalTimer & if\;Traffic\;\;type=NRTT \end{array}\right. \end{array}$$ (11)

If the traffic type is real time traffic, the system will use Real Time Traffic timer (*RTT*_*t*_*imer*). The delay requirement for real time traffic is provided with the packets QoS parameters. The packet maximum acceptable transfer time over the network can be determined by *Max*_*Delay*_. In CBR and VBR traffic, the packet maximum transfer time over the network is clearly stated in a parameter called Maximum Cell Transfer Delay (MaxCTD). Thus, to fulfil the real time traffic QoS delay requirements over OBS, the *Max*_*Delay*_ parameter must not be less than the OBS entire delay, i.e.,

$$\begin{array}{c} Max_{Delay}\geq OBS_{Entire\_Delay} \end{array}$$ (12)

In the OBS network, the entire delay is the collection of the Burst Assembly time *T*_*out*_, the Offset Time (OT), and the Propagation delay (*P*_*d*_*elay*).

$$\begin{array}{c} OBS_{Entire\_Delay}=T_{out}+OT+P_{delay} \end{array}$$ (13)

However, to guarantee the real time traffic end to end delay required, the delay in other domains must be considered. Thus it is assumed that, Other domains Delay *O*_*Delay*_ value is variable such that it can be used for any delay in other domains than OBS, and *p* is any potential value. As a result, the *Max*_*Delay*_ must be greater than or equal to these parameters, so,

$$\begin{array}{c} OBS_{Max\_Delay}\geq OT\;+T_{out}\;+P_{delay}\;+O_{Delay}\;+p \end{array}$$ (14)

In the OBS networks, *P*_*delay*_ is calculated based on the distance between the source and the destination *(d)* and the wavelength propagation speed *(s)* as follows:

$$\begin{array}{c} P_{delay}=\frac{d}{s} \end{array}$$ (15)

While the offset time is calculated based on number of nodes between the source and the destination, as well as the switching time of the core node, as follows:

$$\begin{array}{c} OT=h\times PT\times ST \end{array}$$ (16)

where *h* is the number of nodes between the source and the destination, *PT* is the processing time and *ST* is the switching time of the core node. By compensating Eqs (16) and (15) in Eq (14), the maximum delay, *Max*_*Delay*_, can be written as Eq (17).

$$\begin{array}{c} Max_{Delay}\geq \left(h\times PT+ST\right)+T_{out}+O_{Delay}+p\;+\frac{d}{s}\;\left(17\right) \end{array}$$ (17)

To fit the *Max*_*D*_*elay* value with the OBS entire delay as in the previous equation, either the offset time value or the time out value must be adjusted. As stated earlier, the offset time is calculated based on *h*, and *ST*. Changing any of the offset time values requires a faster and costlier optical switching technology. As a result, the value of the *T*_*out*_ is nominated to be arranged to guarantee the *Max*_*Delay*_ requirements. Hence, Eq (17) is expressed by:

$$\begin{array}{c} T_{out}\leq Max_{Delay}-\left(h\times PT+ST+O_{Delay}+p\right)+\frac{d}{s} \end{array}$$ (18)

Observing Eq (18), choosing the appropriate value for the burst assembly *T*_*out*_ parameter will lead to fulfil QoS delay requirements and traffic delay contract. Consequently, the value of the *MaxCTD* is to be checked for the coming data to determine the delay requirements of the real time traffic as follows:

$$\begin{array}{c} Max_{Delay}\{\begin{array}{cc} MaxCTD, & if\;MaxCTD<Max_{Delay}\\ Max_{Delay}, & otherwise \end{array} \end{array}$$ (19)

As can be seen from Eq (19), if the new value of *MaxCTD* is less than the current temporary delay requirements (*Max*_*delay*_), then the system will change the *Max*_*delay*_ value to the new required one, which is the new *MaxCTD* value. Besides, RT-QoSFR will aggregate and send the current burst which was configured based on the previous delay requirements to ensure that each data packet has been met with its delay requirements. Then, the value of the Real Time Traffic timer (*RTT*_*timer*_) is calculated based on the new value as follows:

$$\begin{array}{c} RTT_{timer}=Max_{Delay}-\left(h\times PT+ST+O_{Delay}+p\right)+\frac{d}{s} \end{array}$$ (20)

The real time traffic timer value after that is assigned to the *T*_*out*_,

$$\begin{array}{c} T_{out}=RTT_{timer} \end{array}$$ (21)

On the other hand, if the new value of *MaxCTD* is not less than *Max*_*delay*_, then the system continues with the previous delay requirements. Afterwards, all packets that arrived during this period will be queued at the destination queue. RT-QoSFR checks whether the *T*_*out*_ or *B*_*max*_ reaches its maximum value to send the burst, it will determine the network traffic load to adapt the data burst aggregation ratio according to the network traffic load and send the data burst. After getting the traffic type, RT-QoS distinguishes the network traffic load into three categories according to the network load which are the high load, normal load, and low load. Based on the network load, RT-QoSFR scheme will adapt the ratio of the real time traffic inside the burst. The ratio of the real time traffic packets will be set to an initial ratio plus membership value for every category. After obtaining the network traffic load rates, RT-QoSFR will determine the traffic load category to be high, normal, or low traffic load. Then, RT-QoSFR will find the Membership value (*M*_*Value*_) for each traffic load category as follows:

$$\begin{array}{c} M_{Value}\{\begin{array}{cc} \frac{L_{avg}-H_{load}}{\left(100-H_{load}\right)/A_{range}}, & if\;load=high\\ \frac{L_{avg}-L_{load}}{\left(H_{load}-L_{load}\right)/A_{range}}, & if\;load=normal\\ \frac{L_{avg}}{L_{load}/A_{range}}, & if\;load=low \end{array} \end{array}$$ (22)

where, *L*_*a*_*vg* determines the network traffic load rate per 1 second, *H*_*load*_ represents the value of the beginning (minimum value of high traffic load) of the high traffic load, *A*_*rang*_ stands for the maximum value of the membership value, and *L*_*load*_ determines the highest value of the low traffic load parameters. After that, RT-QoSFR will find the Real Time Traffic average (*RTT*_*avg*_) in the burst by adding the *M*_*Value*_ to the ratio of real time traffic inside the burst based on the network traffic load. So, the *RTT*_*avg*_ will be calculated as,

$$\begin{array}{c} RTT_{avg}\{\begin{array}{cc} (H_{avg}+M_{Value}), & if\;load=high\\ (Low_{avg}+M_{Value}), & if\;load=low\\ (N_{avg}+M_{Value}), & if\;load=normal \end{array} \end{array}$$ (23)

where, *H*_*avg*_, *Low*_*avg*_ and *N*_*avg*_ determine the ratios of real time traffic inside the burst for high between (50—60%), low between (10—20%) and normal between (30—40%) traffic load parameters, *A*_*range*_ stands for the maximum value of the membership value. After getting the average of real time traffic in the burst, RT-QoSFR will find the total number of bytes for the real time traffic (*RTT*_*Bytes*_) and the non real time traffic (*NRTT*_*Bytes*_) in the burst as the following,

$$\begin{array}{c} RTT_{Bytes}=B_{size}\times RTT_{avg}/100 \end{array}$$ (24)

$$\begin{array}{c} NRTT_{Bytes}=B_{size}-RTT_{Bytes} \end{array}$$ (25)

where *B*_*size*_ is the summation of the Burst size. The next step, RT-QoSFR will aggregate the burst with *RTT*_*Bytes*_ from the real time traffic queue and *NRTT*_*Bytes*_ from the non-real time traffic queue. Finally, the system will check if there is a continuous flow of data. If this happens, the system will repeat the same procedures.

**Fig 7:**
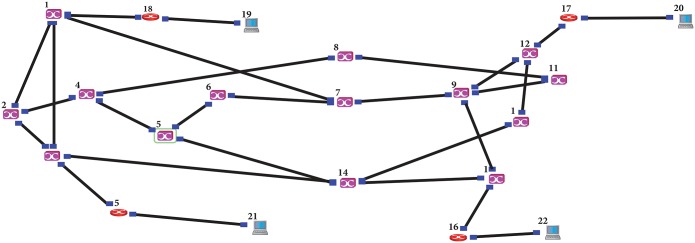
The process flow of RT-SQFR scheme.

**Fig 8:**
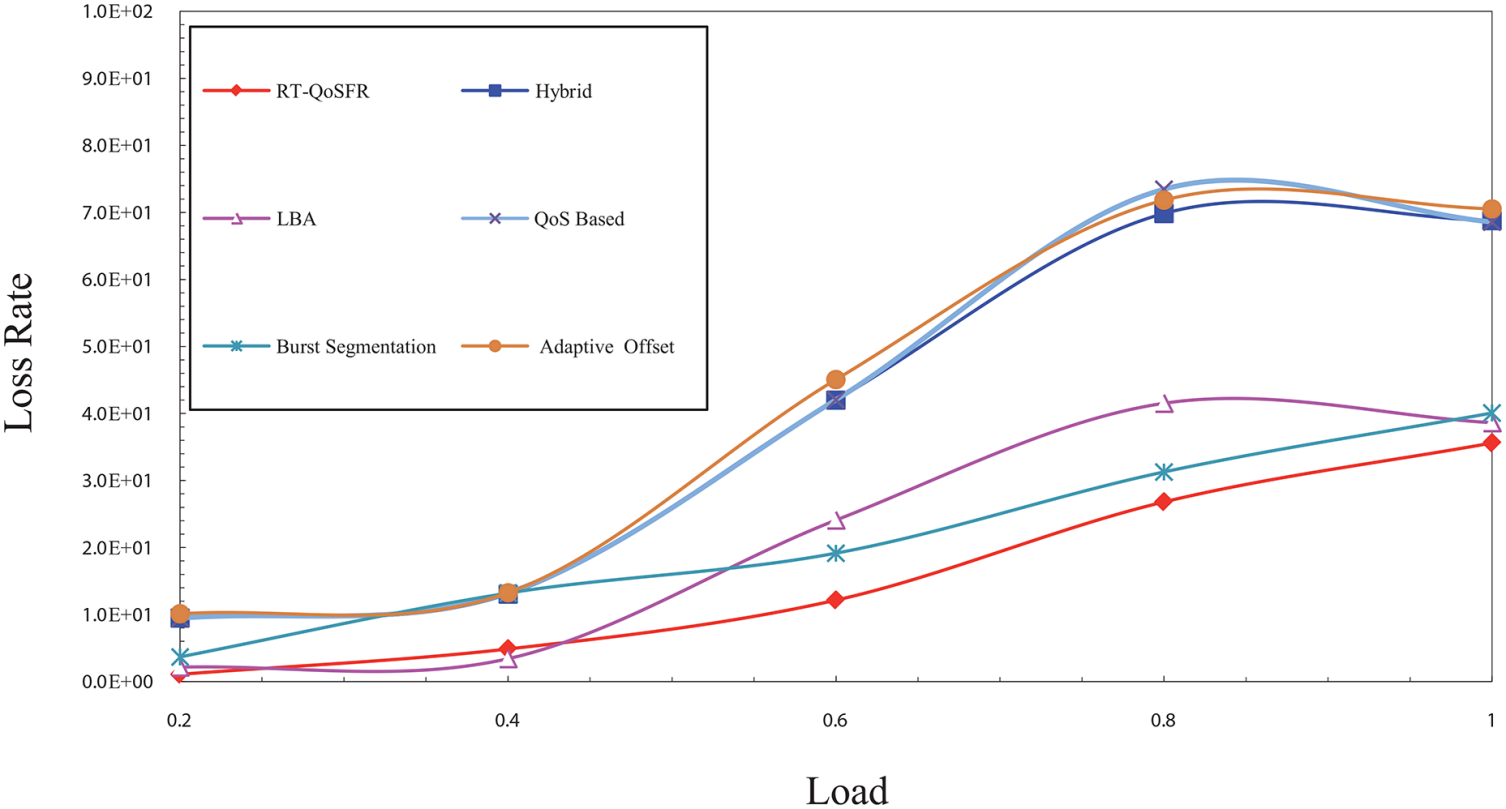
Phase 1 of the process flow of RT-SQFR scheme.

**Fig 9:**
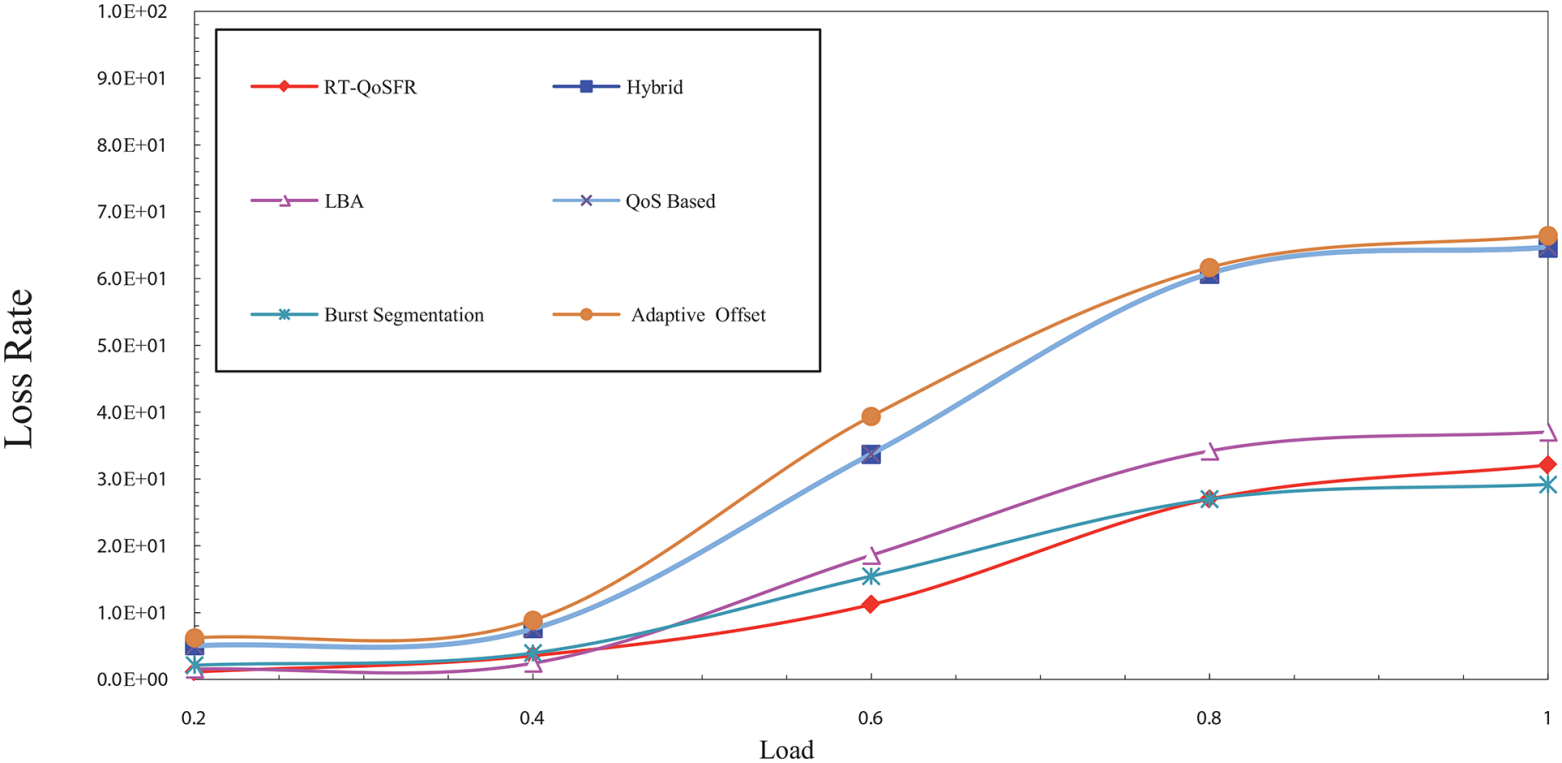
Phase 2 of the process flow of RT-SQFR scheme.

**Fig 10:**
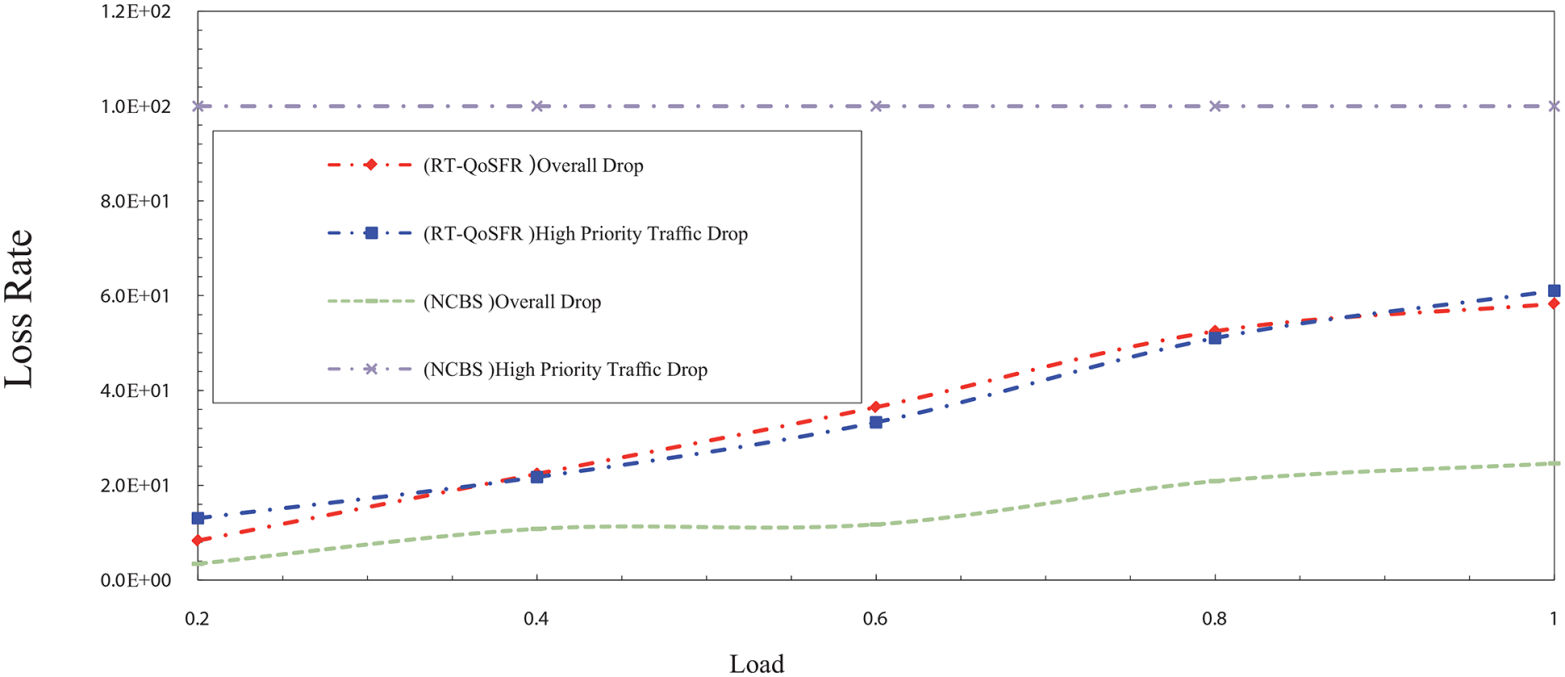
Phase 3 of the process flow of RT-SQFR scheme.

Fig 7 shows the flow chart that describes the RT-SQFR scheme. The system is divided into three phases (Figs 8, 9 and 10) that are network traffic identifier, network traffic delay controller, and network traffic classifier and assembler controller.

### Simulation Scenarios

**Fig 11:**
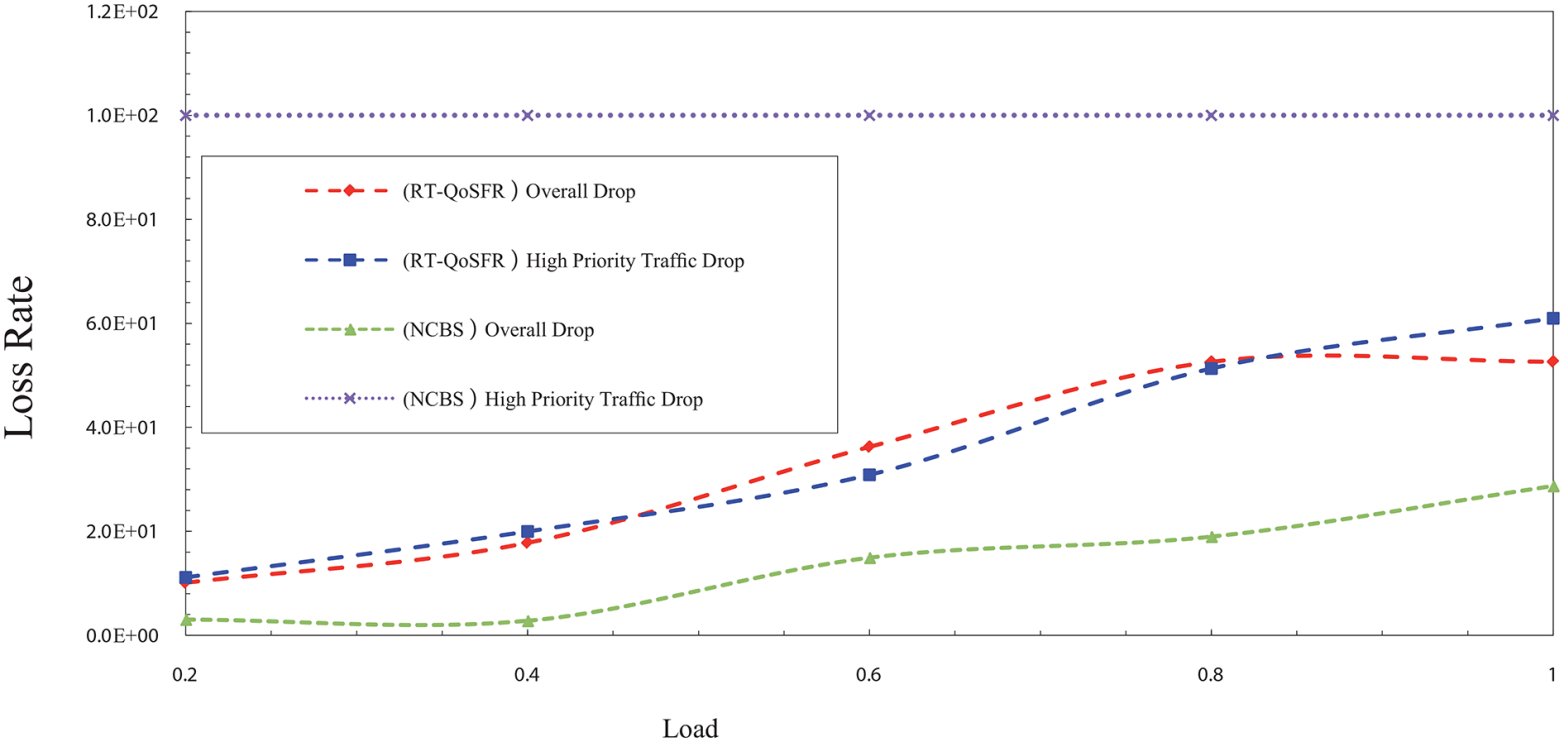
Four node OBS topology.

**Fig 12:**
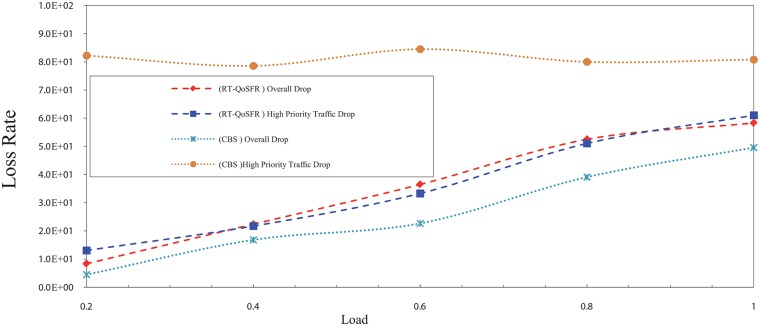
NSFNET Topology.

In this section, the simulation scenarios that have been used in this paper are introduced. The simulation has used NCTUns simulator to develop, evaluate the performance of proposed schemes and compare them with other schemes. The proposed schemes have been studied using the simulation model with two types of traffic (CBR, VBR), four values of MaxCTD, two value of burst size, and two different topologies (the simple OBS topology is shown in Fig 11, and the NSFNET topology is shown in Fig 12). The objective of these scenarios is to demonstrate the possibility of the proposed scheme to work under various conditions.

The configuration of the simulation models is divided into two parts: the OBS network configuration and the real time traffic configuration. The simulation parameters of the OBS network configuration for both topologies are described in Table 2.

**Table 2:** Simulation Parameters of the OBS Network Configuration.

Parameter	Value
Link bandwidth	1000 Mb/s
Propagation delay	1 *μ* s
Bit error rate	0
Maximum burst length	16000 Byte – 32000 Byte
Use of Fiber Delay Line (FDL)	No
Use of Wavelength Conversion	No

In the real time traffic configuration, CBR traffic and VBR traffic trace files have been created with several traffic load rates as follows: increasing load, high load (maximum bandwidth of the simulator), low load (512kb), and bursty load. BARDR scheme has been studied under all the possible traffic load rates as mentioned above to ensure the guarantee of real traffic delay requirements within any load. Table 3 shows the traffic rate for each assigned load.

**Table 3:** Simulation Parameters of the OBS Network Configuration.

Load Type	Load Rate
Incremental	0.5 Mb – 1000 Mb
High	1000 Mb
Low	0.5 Mb
Bursty	0.5 Mb – 1000 Mb

For all traffic loads, three MaxCTD values have been assumed to be 92 *μ*s, 100 *μ*s, 70 *μ*s, and 125 *μ*s. These values have been assumed in this range in order to get an excellent quality of data.

## Simulation Results and Discussions

The RT-QoSFR scheme has been evaluated using a customized simulation model with several traffic loads, two types of traffic (Constant Bit Rate (CBR), Variable Bitrate (VBR)), and NSFNET topology as shown in Fig 12. Moreover, the proposed scheme has been compared with several schemes such as hybrid scheme, QoS offset-based scheme, adaptive offset time scheme, and burst segmentation scheme.

**Fig 13:**
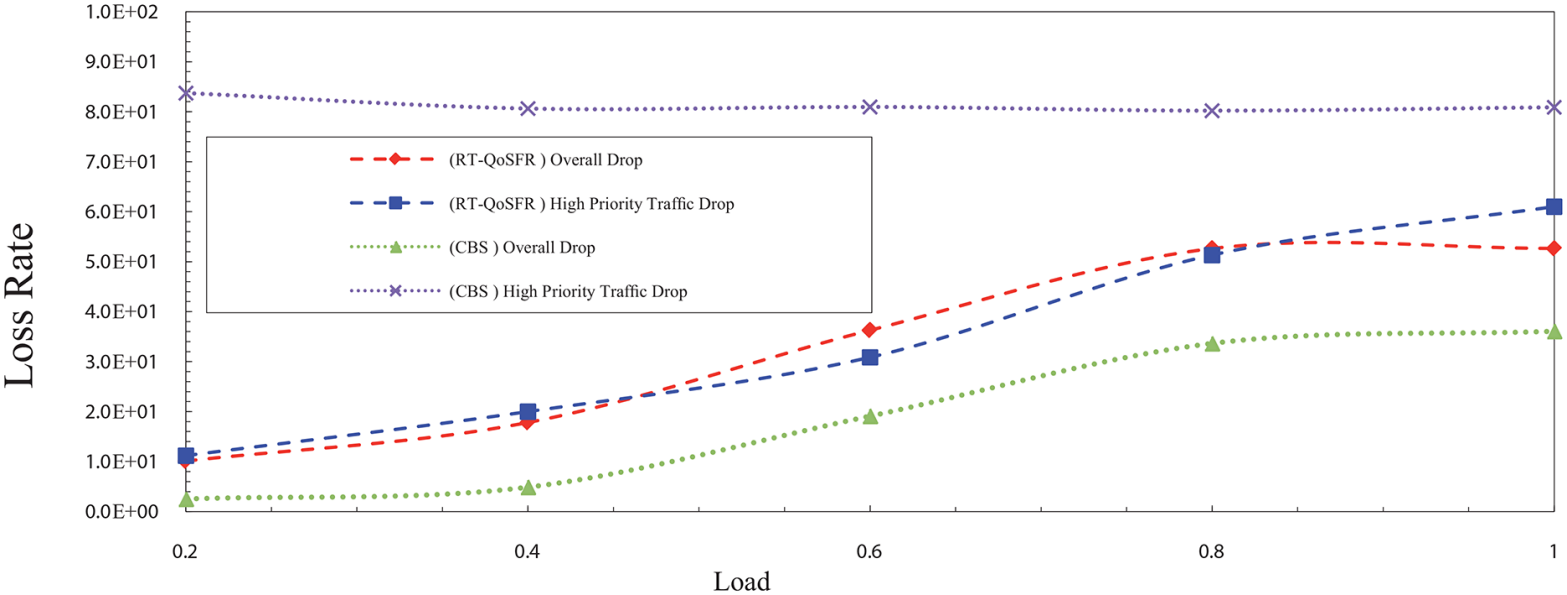
The packet loss probability comparison between RT-QoSFR and other schemes using CBR traffic.

Fig 13 shows the packet loss probability comparison between RT-QoSFR and the other schemes using CBR traffic. The packet loss probability comparison evaluates the real time traffic packets loss. The results show that RT-QoSFR provides a better performance and reduces the real time traffic packet loss compared with the other schemes, which show a high packet loss probability. Moreover, RT-QoSFR shows stability in the performance of the network and ensures the fairness between real time traffic and non real time traffic.

It can be noted from Fig 13 that the packet loss probability of the hybrid, QoS offset-based and burst segmentation schemes is much higher than other schemes. This high packet loss probability may be due to the design of these schemes, in which no adaptive mechanism to reduce the drop is employed. On the contrary, the packet loss probability for RT-QoSFR, LBA and adaptive offset schemes are less due to the use of adaptive mechanisms that reduce the drop based on some rules. However, RT-QoSFR scheme provides a better network performance due to that it considers the fairness issues among the traffic, unlike other schemes where the fairness for the low priority traffic is absent in their design.

**Fig 14:**
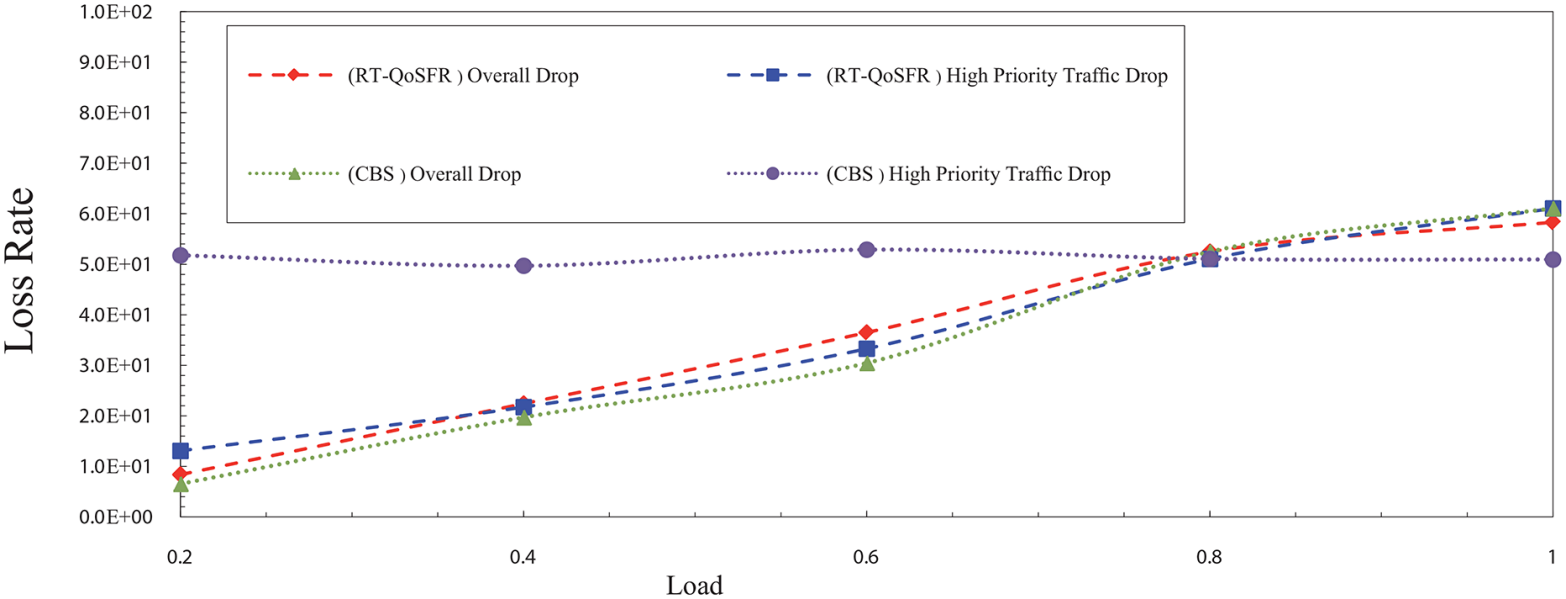
The packet loss probability comparison between RT-QoSFR and other schemes using VBR traffic.

Observing Fig 14, both VBR and CBR traffic exhibit similar results. However, it can be noted that there are some differences in values between VBR traffic and CBR traffic, which are dating back to the nature of VBR traffic that generates packets with different size causing the packet delay to be less. Furthermore, due to that the packets are with different sizes, the probability of the data loss seems different.

In order to show the ability of RT-QoSFR to ensure the fairness for non-real time traffic, RT-QoSFR has been compared with Composite Burst Segmentation (CBS) and Non-CBS (NCBS) for several real time traffic ratios of 80, 50, and 20%. The burst segmentation scheme has been chosen in this comparison due to the results shows that it is the second lowest loss rate in the previous comparison. The results also show that RT-QoSFR scheme guarantees the fairness for non real time traffic packets.

**Fig 15:**
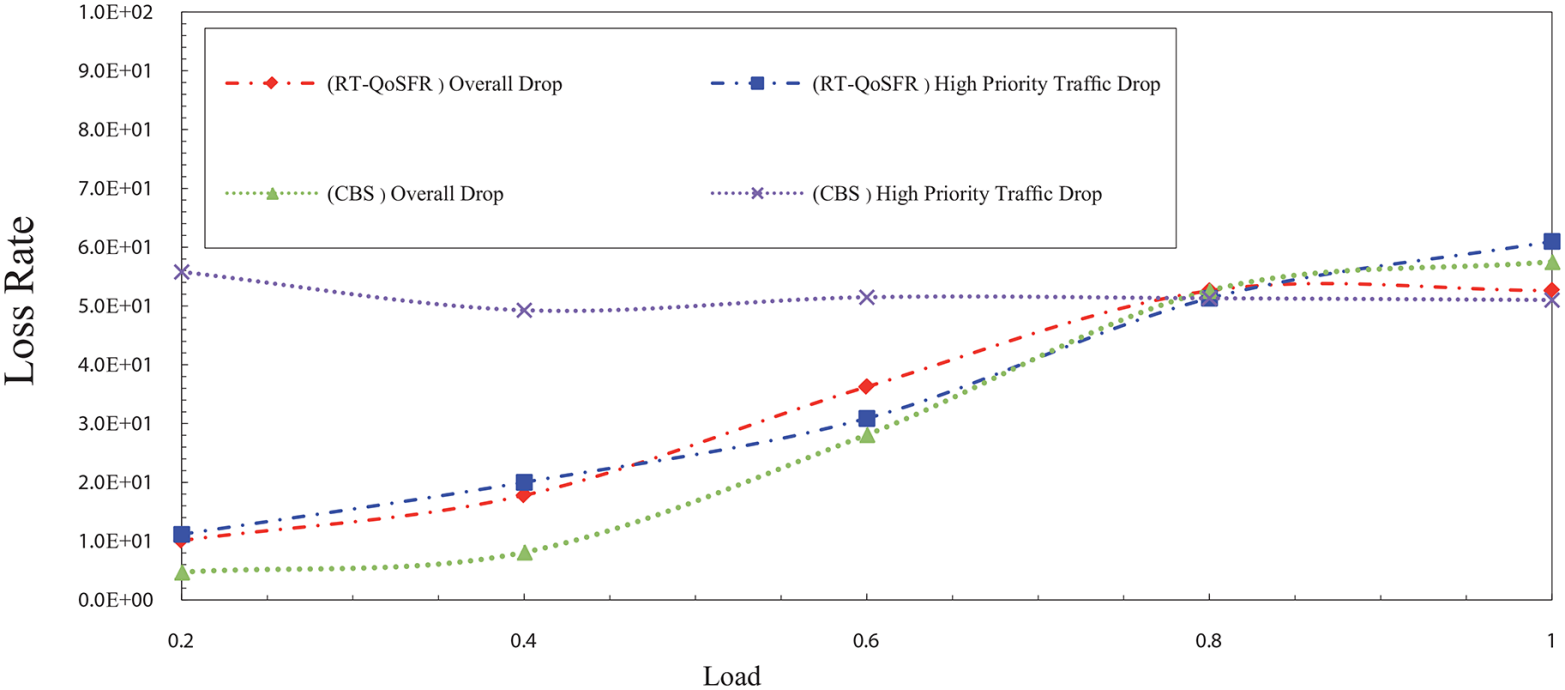
The comparison of packet loss rate between RT-QoSFR and NCBS using CBR traffic.

**Fig 16:**
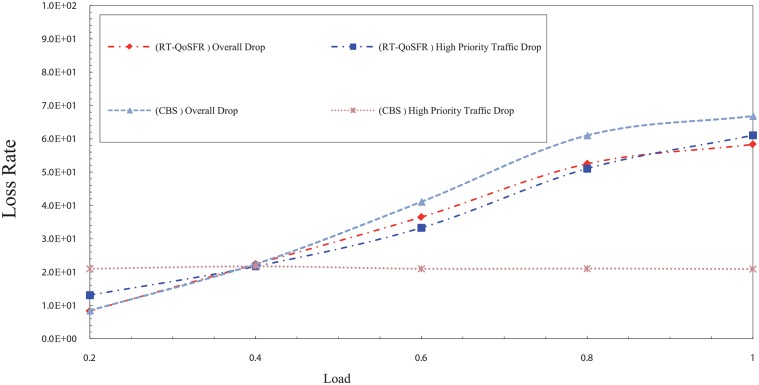
The comparison of packet loss rate between RT-QoSFR and NCBS using VBR traffic.

In the NCBS scheme, the results show that RT-QoSFR can guarantee the real time traffic packet loss, at the same time guarantee the fairness for non real time traffic packets, which lead to the network performance stability. Figs 15 and 16 show the packet loss rate comparison between RT-QoSFR and NCBS using CBR and VBR traffic.

The packets loss rate comparison evaluates the overall packets loss of the network, and the real time traffic packets loss for RT-QoSFR and NCBS schemes. The results clarify that the NCBS scheme reduces the overall loss in general. However, it does not guarantee the packet loss for the real time traffic which is high.

**Fig 17:**
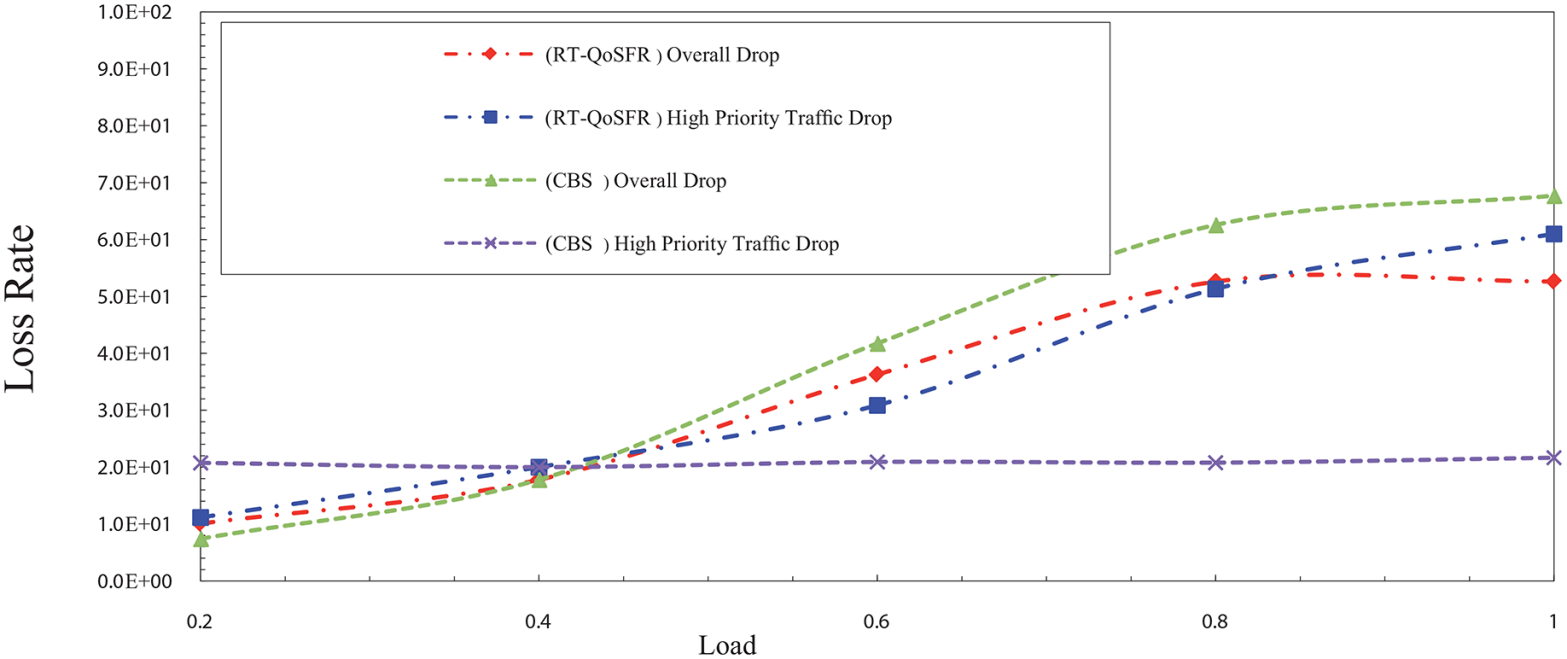
The comparison of packet loss rate between RT-QoSFR and CBS using CBR traffic with 80% real time traffic ratio of the data burst.

**Fig 18:**
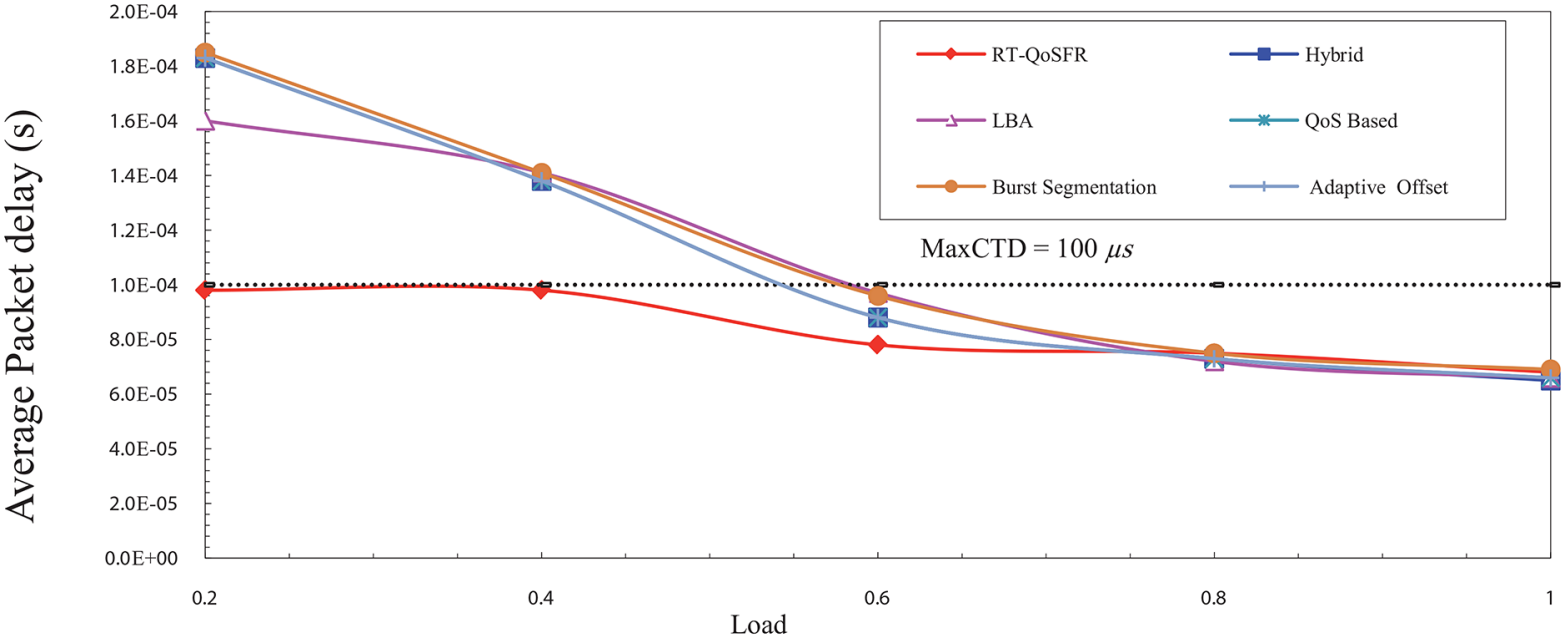
The comparison of packet loss rate between RT-QoSFR and CBS using VBR traffic with 80% real time traffic ratio of the data burst.

**Fig 19:**
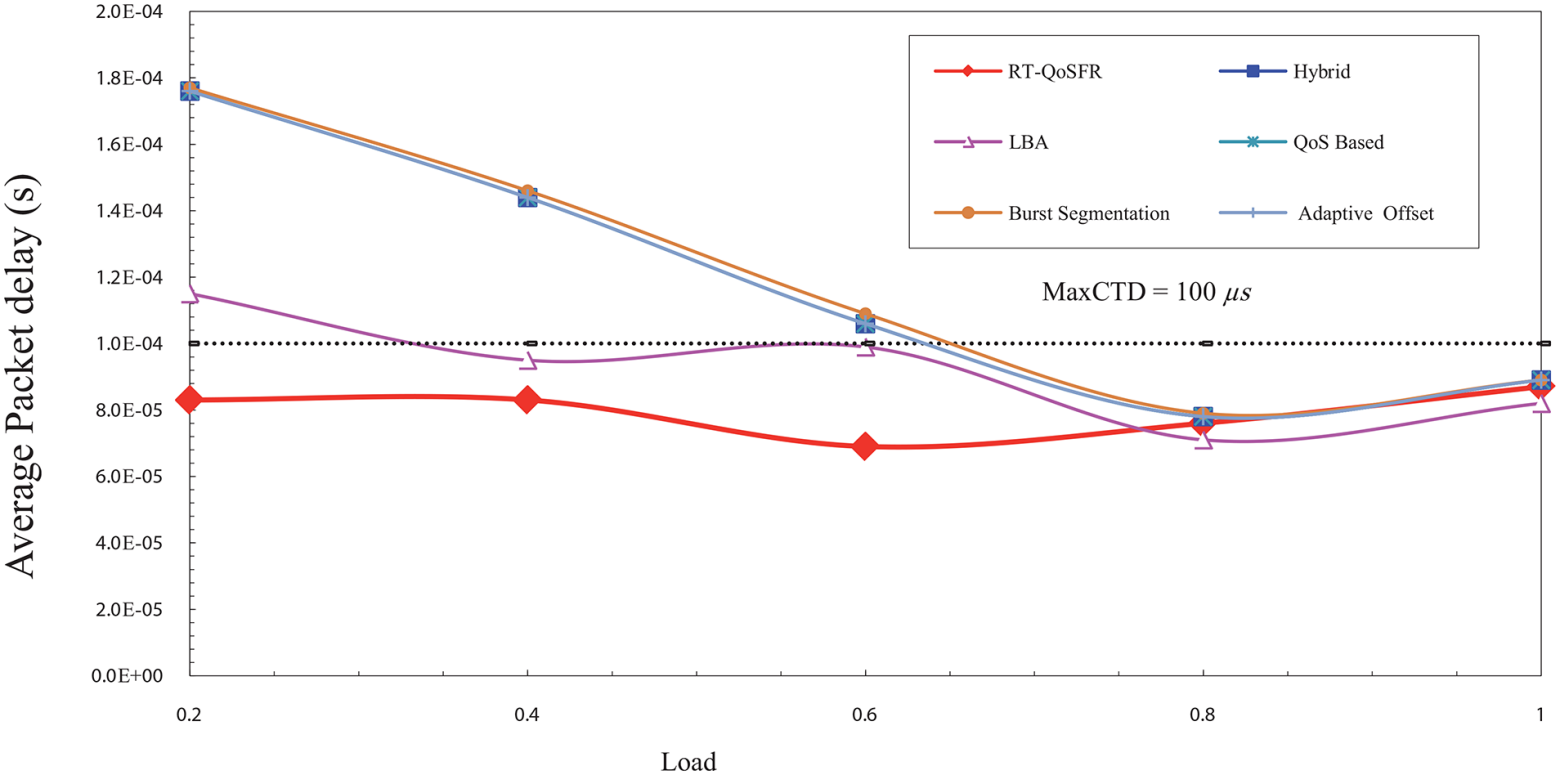
The comparison of packet loss rate between RT-QoSFR and CBS using CBR traffic with 50% real time traffic ratio of the data burst.

**Fig 20:**
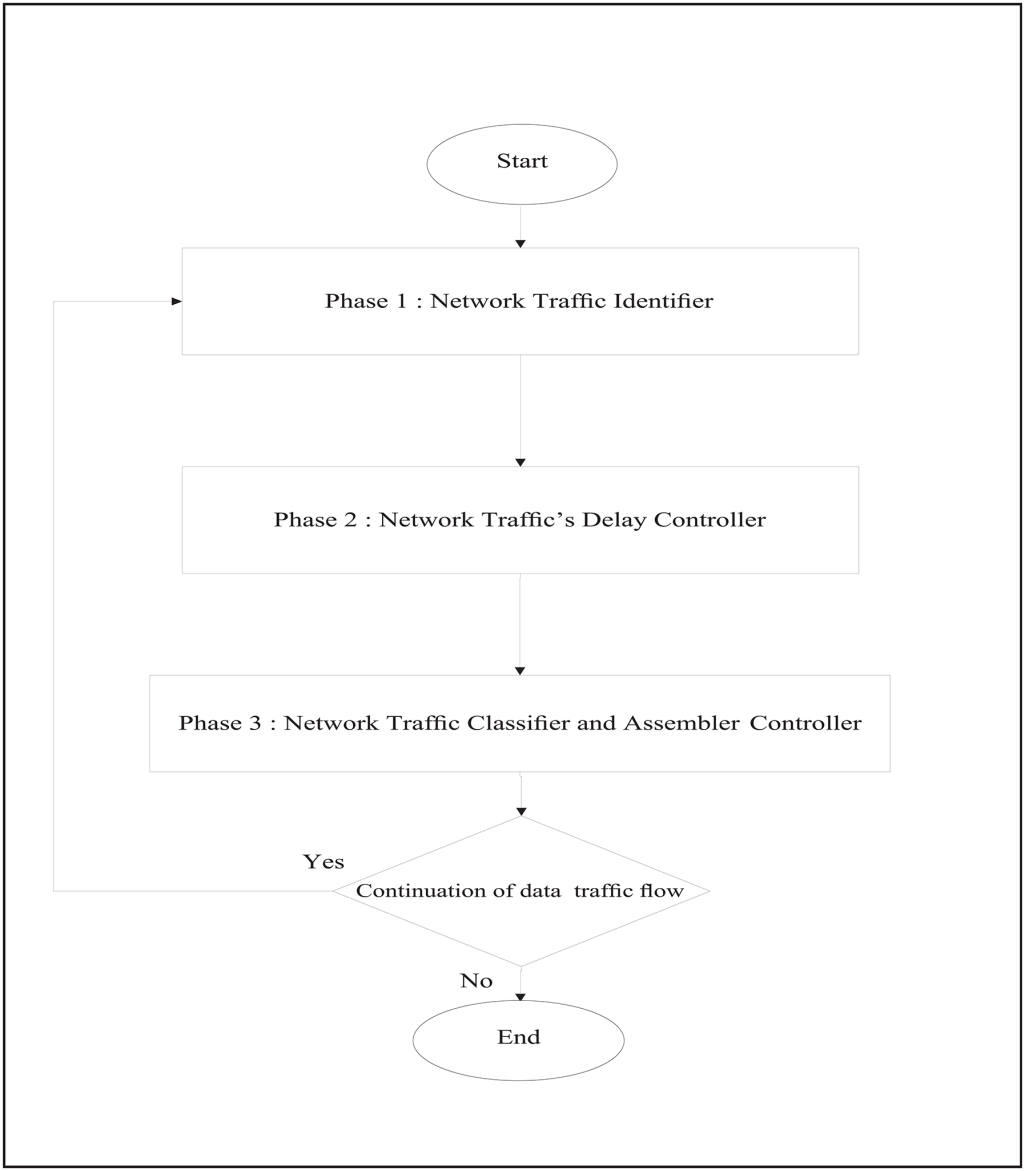
The comparison of packets loss rate between RT-QoSFR and CBS using VBR traffic with 50% real time traffic ratio.

Figs 17, 18, 19 and 20 depict the packet loss rate comparison between RT-QoSFR and CBS using CBR and VBR traffic with 80% and 50% real time traffic ratios. The results demonstrate that RT-QoSFR is able to keep the stability in the performance of the network and ensure the fairness between real time traffic and non real time traffic, which provides a better QoS and reduces the real time traffic packet loss.

**Fig 21:**
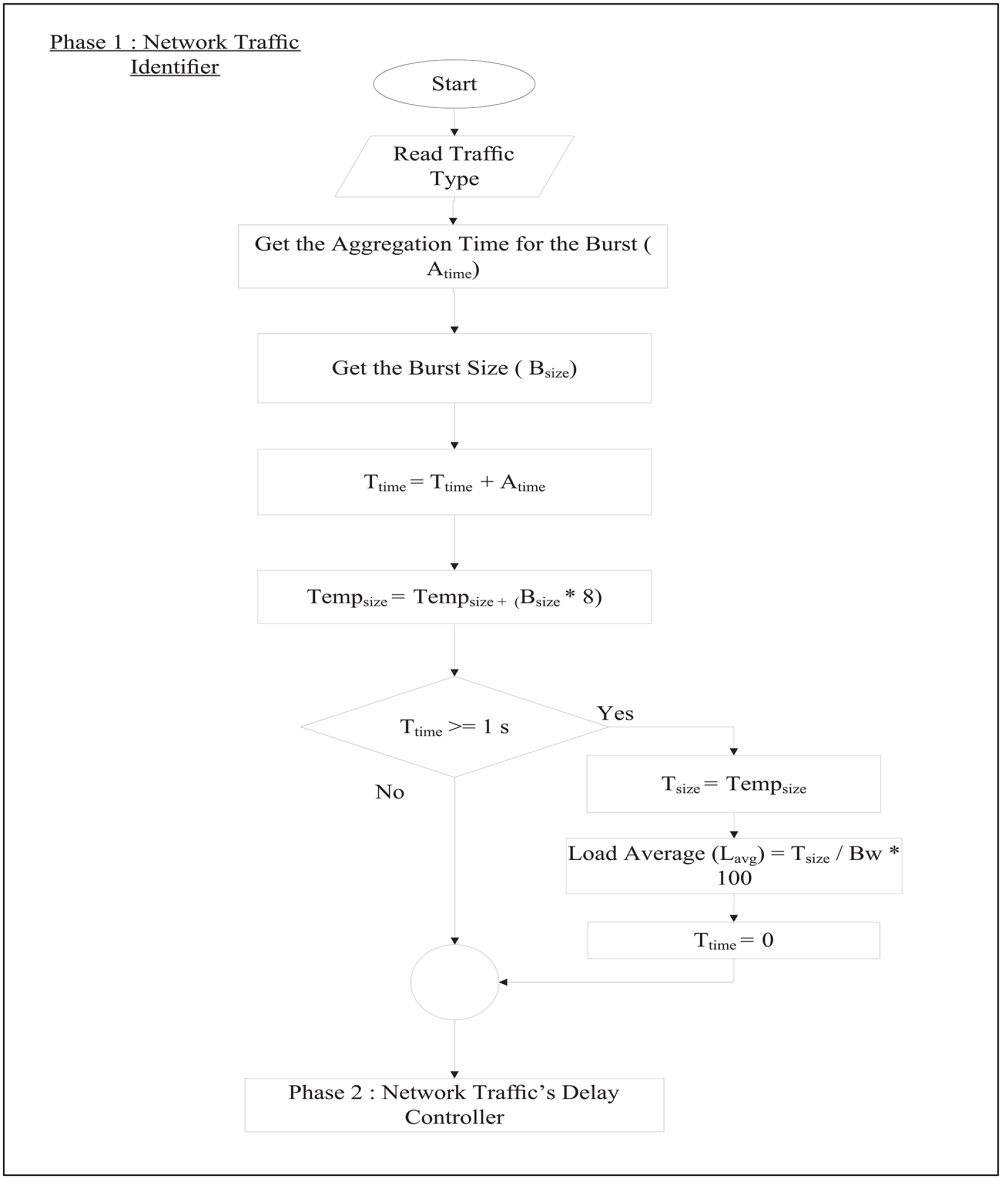
The comparison of packet loss rate between RT-QoSFR and CBS using CBR traffic with 20% real time traffic ratio.

**Fig 22:**
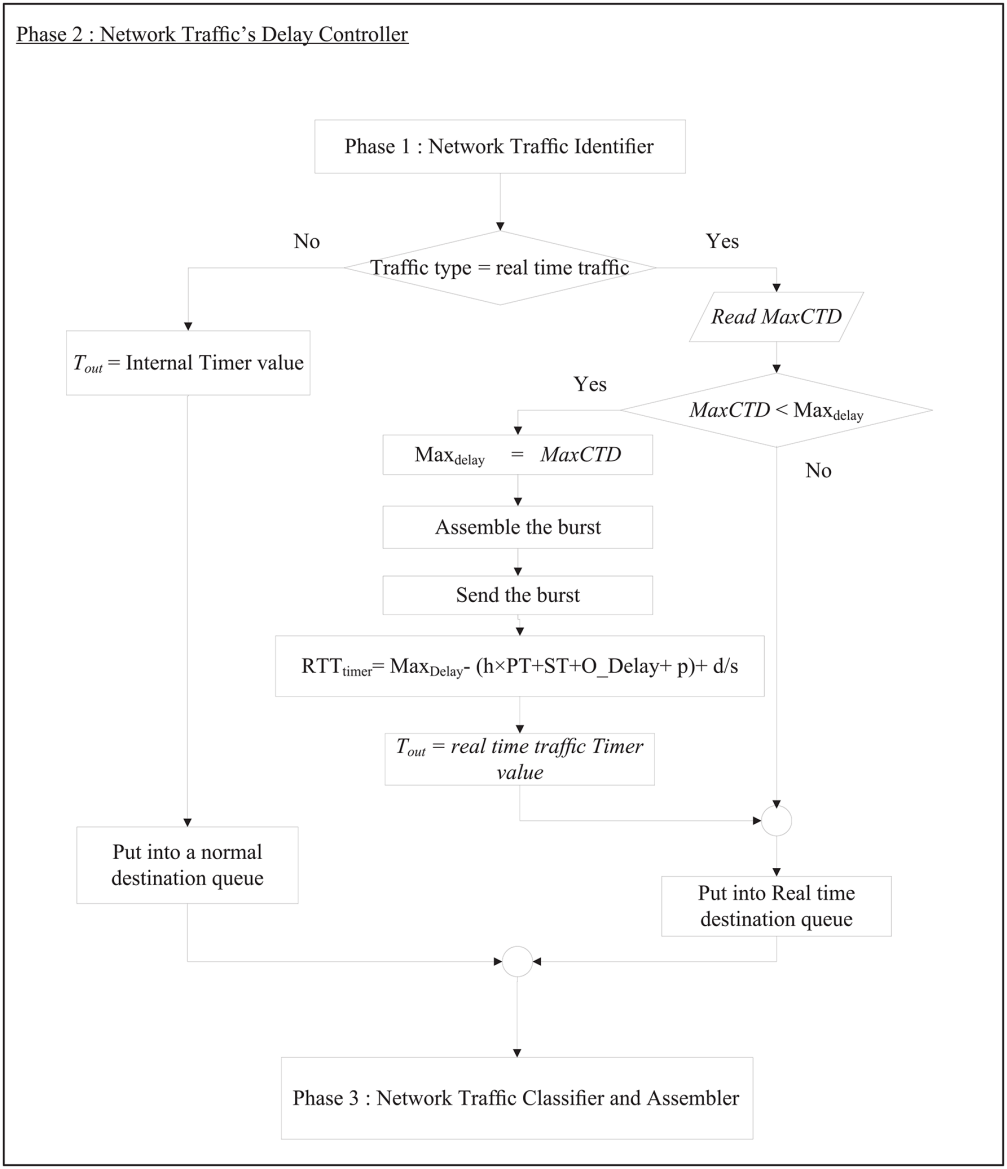
The comparison of packet loss rate between RT-QoSFR and CBS using VBR traffic with 20% real time traffic ratio of the data burst.

On the other hand, the results in Figs 21 and 22 illustrate that a real time traffic ratio of 20% can reduce the real time traffic packets rate. However, the overall packet loss rate is high, which leads to increase the number of lost packets from the real time traffic, as well as to fluctuate the network performance stability.

In contrast, the results in RT-QoSFR series show that the rate of loss for real time traffic packets is higher than CBS in the case of 20%. However, the stability in the performance of the network and the fairness among real time traffic and non real time traffic leads to provide a better QoS to the real time traffic packets. Moreover, the overall packet loss rate in RT-QoSFR series is less than CBS in the case in 20%. This also reduces the total number of lost packets from the real time traffic.

For the real time traffic delay requirements, the results show that the entire delay using RT-QoSFR does not exceed the *Max*CTD value unlike the other schemes, which exceed the *Max*CTD value in the low load rate. Moreover, it shows that RT-QoSFR scheme guarantees the traffic delay requirements in all traffic load cases. Furthermore, it guarantees the delay requirements, even in the presence of a contention topology.

**Fig 23:**
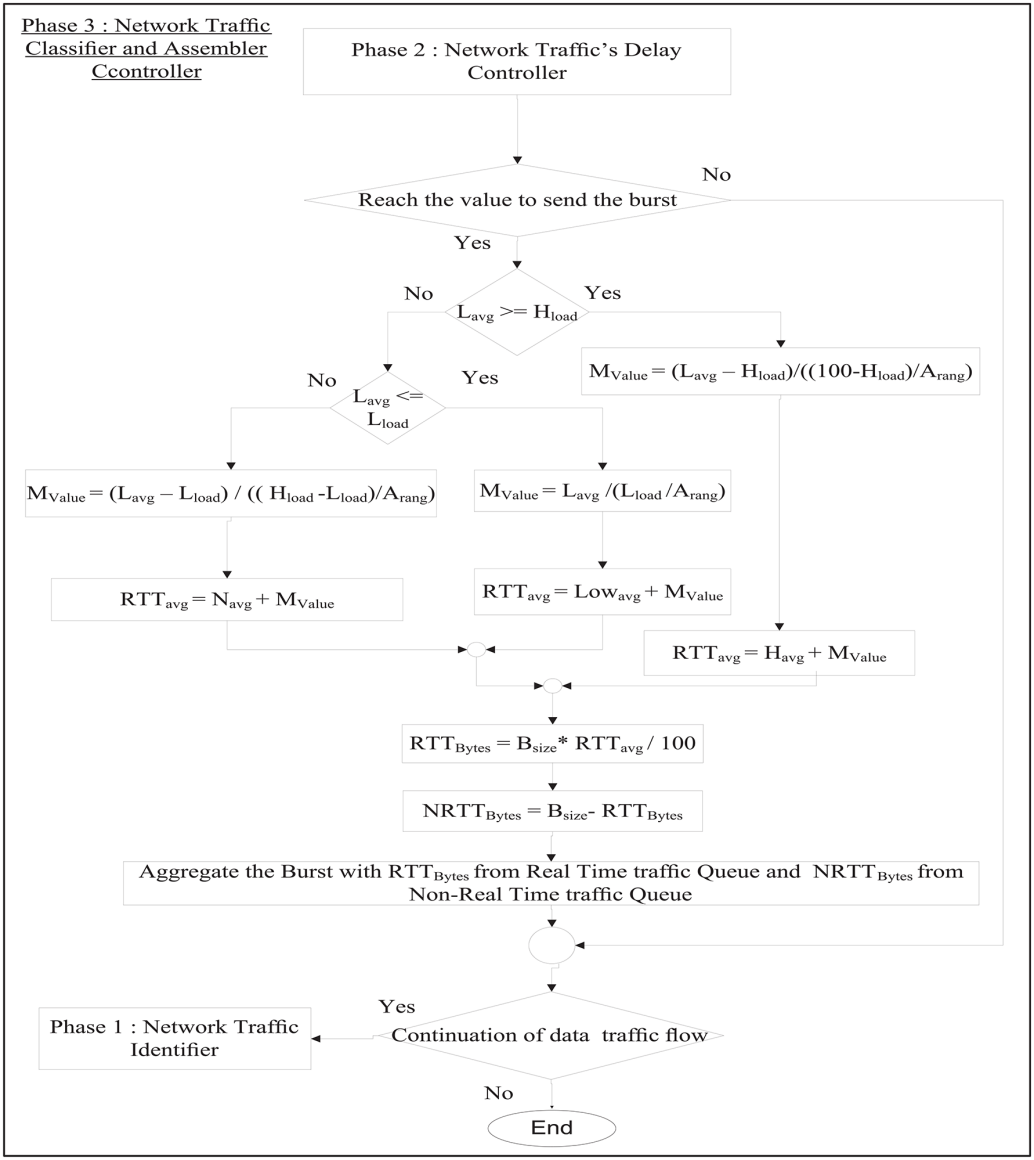
The average CBR packet delay comparison among the schemes using incremental load rate.

Fig 23 depicts the packets delay comparison among the schemes under increasing load rate. The rate of sending the data is incremental where the low load points start at 10% and increase in each point up to 49%. While the high load starts at 50% and increases in each point up to 100%.

In the low offered load, it is noticed that the RT-QoSFR scheme delay is less than the *Max*CTD value, which equals to 100 *μ*s. This value represents the maximum packet delay in this simulation model. On the other hand, the average packet delays for other schemes are exceeding the *Max*CTD value for the other schemes. This is because the rate of low load traffic, normally, is not enough to create a burst within the interval time specified in the timer. Consequently, the data packets have to wait in order to aggregate enough data or until the *T*_*out*_ parameter reaches its maximum value, this leads not to fulfil the traffic delay requirements.

RT-QoSFR scheme, in case of low load traffic, can guarantee the packet delay requirements by setting the value of *T*_*out*_ parameter based on the value of *Max*CTD. This process led to allow the data packets to be sent before its maximum delay time. Therefore, for RT-QoSFR scheme the average packet delay does not exceed the maximum packet delay level as shown in Fig 23.

**Fig 24:**
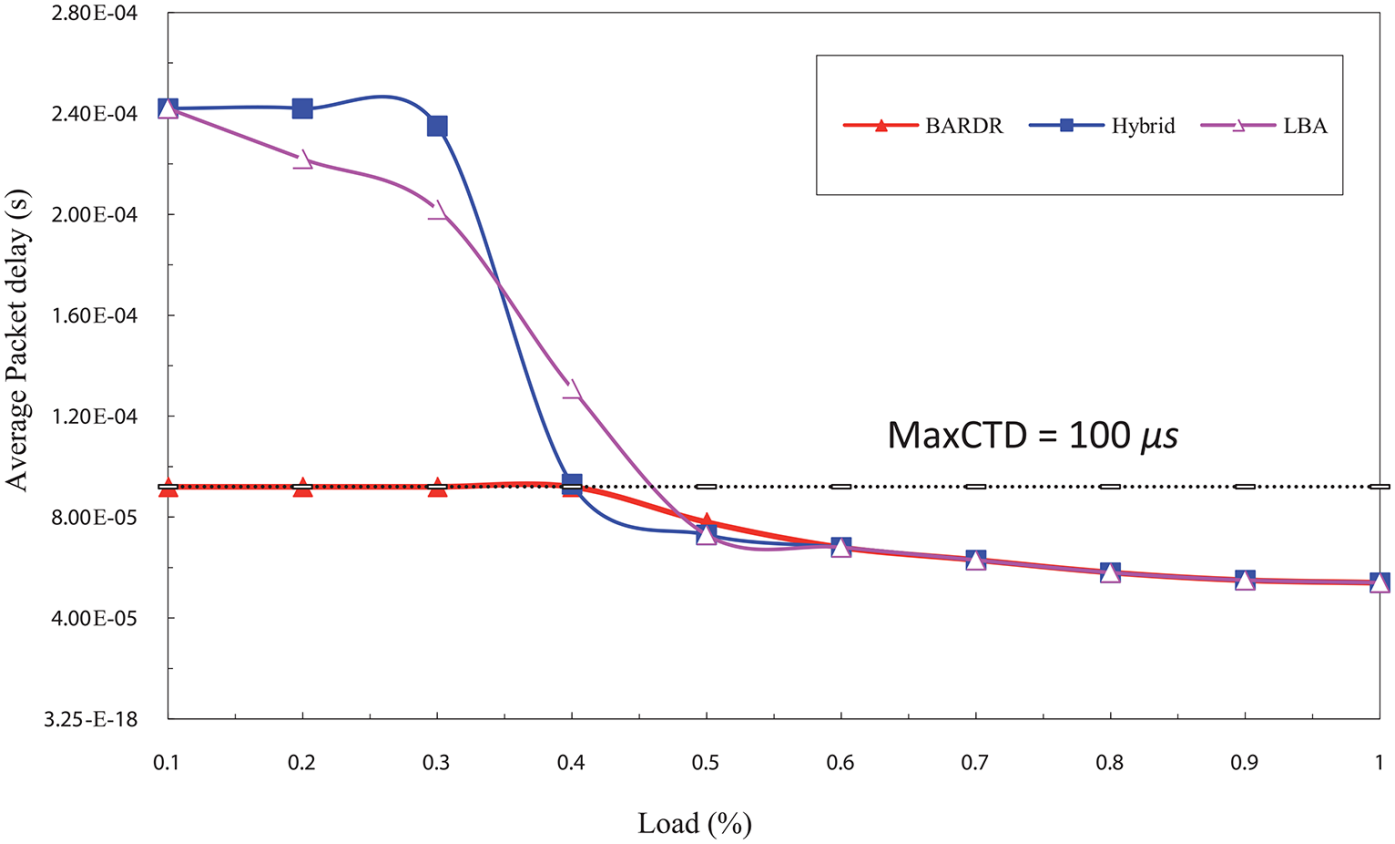
The average VBR packet delay comparison among the schemes using incremental load rate.

In contrast, in the high load traffic, the amount of data is enough to create a burst before the interval timer value reaches its maximum value. Thus, the burst will be sent in a time that is lesser than the maximum packet delay. Consequently, the RT-QoSFR scheme delay curve is convergent with the other schemes because the timers will not work in this case and the burst will be sent based on the amount of traffic, which is already equal. In the case of VBR traffic shown in Fig 24, the results are similar to that obtained in the CBR case. However, it can be noted that there are some differences between VBR traffic and CBR traffic, these differences are dating back to the nature of VBR traffic which comes with different size and burst load that leads to make the packets end to end delay less. Furthermore, due to that the packets are different in size, the values of the time delay are also different.

In the case of CBR traffic, the convergent with the curves of RT-QoSFR and other schemes occurs after the load of 60%. While, in VBR case the convergent starts at the load of 80% due to the different sizes of packets in VBR traffic. Generally, The results show that RT-QoSFR scheme can reduce the real time traffic packets loss, at the same time guarantee the fairness for non real time traffic packets, and guarantee the delay requirements for the real time traffic.

In this section, a simulation model has been developed to study RT-QoSFR scheme with Simple four-node OBS (SOBS) topology to prove the ability of RT-QoSFR scheme to work with various network topologies and nodes.

**Fig 25:**
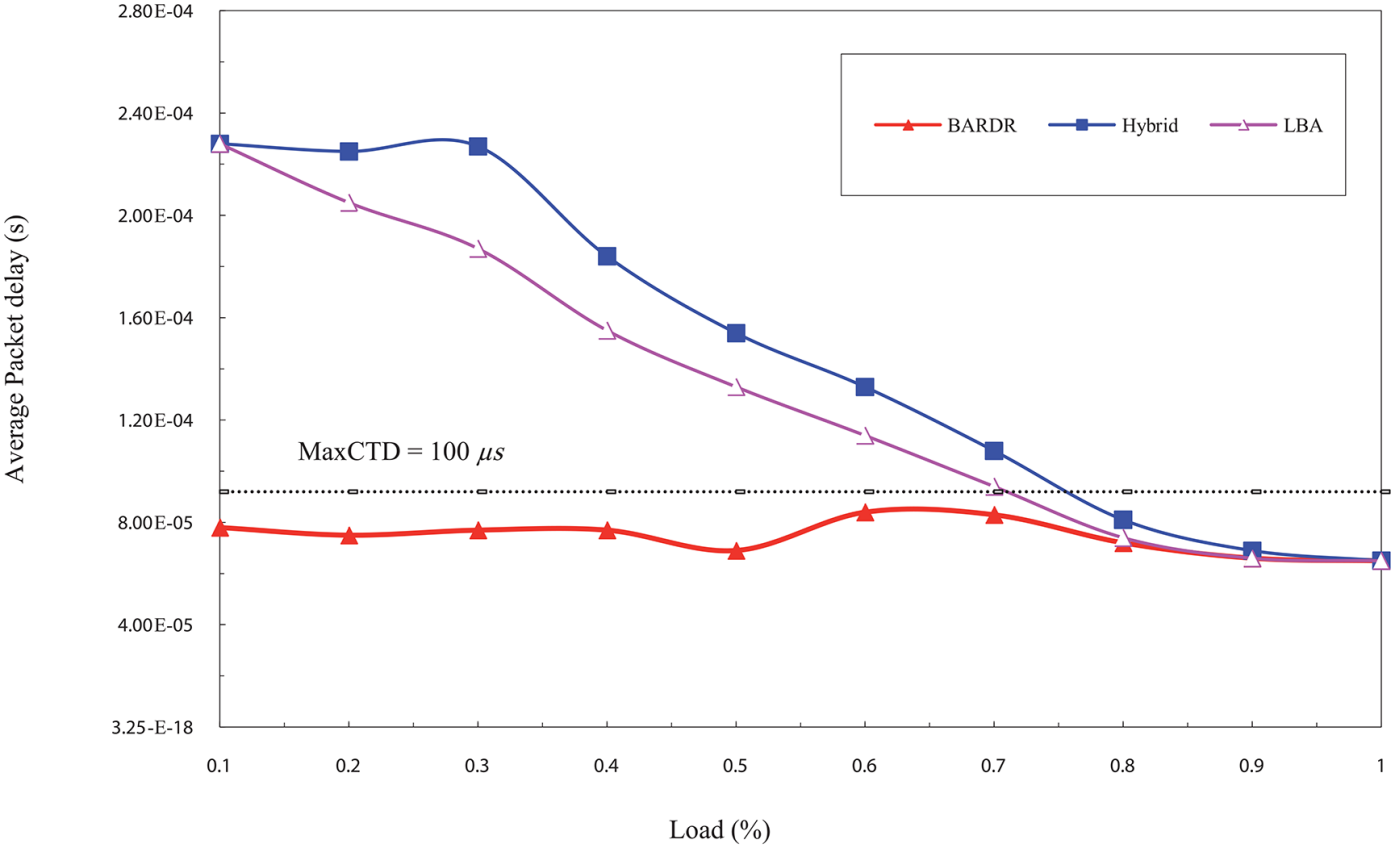
Comparison of CBR average packets delay among the schemes using incremental load rate using SOBS Topology.

**Fig 26:**
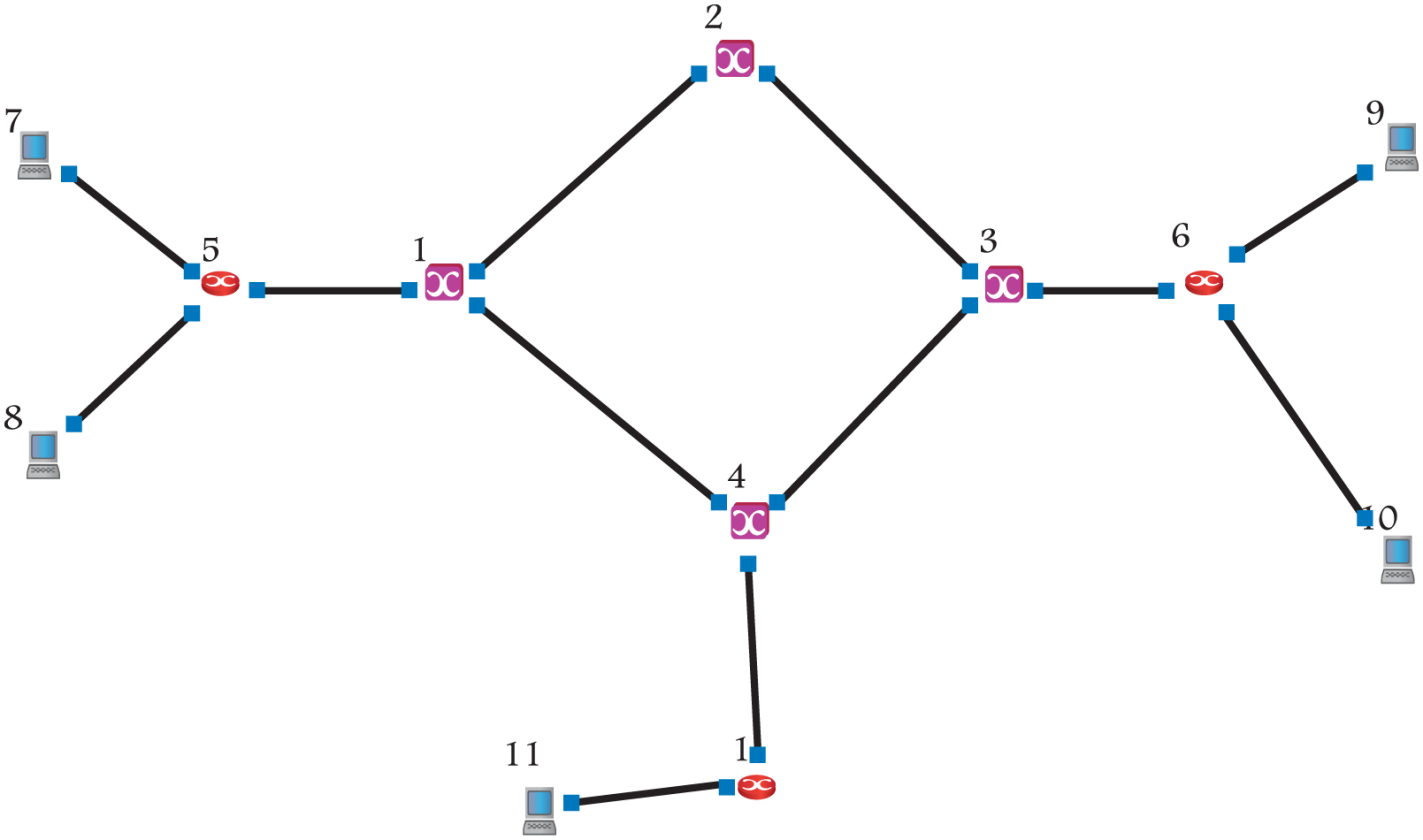
Comparison of VBR average packets delay among the schemes using incremental load rate using SOBS Topology.

The results show that RT-QoSFR scheme is able to guarantee the delay requirements with various network topologies and nodes. It is noted that RT-QoSFR scheme was able to guarantee the average packet delay with the required MaxCTD. The average packet delay does not exceed the value of 100*μs* from the load of 10% to the load 50%. In the high traffic load, the average packet delay is lesser than the required maximum delay. Figs 25 and 26 show comparison of CBR and VBR average packets delay among the schemes using incremental load rate using SOBS Topology.

## Conclusions

This paper has proposed a novel RT-QoSFR scheme that can adapt the burst assembly parameters according to the traffic needs to guarantee the real time traffic requirements and ensure the fairness for the other network traffics.

RT-QoSFR has classified the network traffic load into three categories which is utilized to adapt the ratio of the real time traffic inside the burst for reducing the real time traffic packets loss and guaranteeing the fairness for non real time traffic packets. The fairness ratio for the real time traffic packets in the burst have been found to be 50—60%, 30—40%, and 10—20% for high, normal, and low traffic loads, respectively.

The results show that RT-QoSFR can guarantee the entire delay of OBS network such that it does not exceed the *Max*CTD parameter value in the real time traffic. Furthermore, it can reduce the real time traffic packets loss and guarantee the fairness for non real time traffic packets. Moreover, RT-QoSFR guarantees the stability in the performance of the network, the delay requirements, and ensures the fairness between real time traffic and non real time traffic, which provides a better QoS.
